# Improved Parameterization for the Size Distribution of Emitted Dust Aerosols Reduces Model Underestimation of Super Coarse Dust

**DOI:** 10.1029/2021GL097287

**Published:** 2022-04-19

**Authors:** Jun Meng, Yue Huang, Danny M. Leung, Longlei Li, Adeyemi A. Adebiyi, Claire L. Ryder, Natalie M. Mahowald, Jasper F. Kok

**Affiliations:** ^1^ Department of Atmospheric and Oceanic Sciences University of California Los Angeles CA USA; ^2^ Now at Earth Institute Columbia University New York NY USA; ^3^ Now at NASA Goddard Institute for Space Studies New York NY USA; ^4^ Department of Earth and Atmospheric Sciences Cornell University Ithaca NY USA; ^5^ Now at Department of Life & Environmental Sciences University of California Merced CA USA; ^6^ Department of Meteorology University of Reading Reading UK

**Keywords:** super coarse dust particle, dust particle size distribution, brittle fragmentation theory, CESM, dust modelling, dust emission

## Abstract

Aircraft measurement campaigns have revealed that super coarse dust (diameter >10 μm) surprisingly accounts for approximately a quarter of aerosols by mass in the atmosphere. However, most global aerosol models either underestimate or do not include super coarse dust abundance. To address this problem, we use brittle fragmentation theory to develop a parameterization for the emitted dust size distribution that includes emission of super coarse dust. We implement this parameterization in the Community Earth System Model (CESM) and find that it brings the model in good agreement with aircraft measurements of super coarse dust close to dust source regions. However, the CESM still underestimates super coarse dust in dust outflow regions. Thus, we conclude that the model underestimation of super coarse atmospheric dust is in part due to the underestimation of super coarse dust emission and likely in part due to errors in deposition processes.

## Key Points

- We develop a model parameterization for the size distribution of emitted dust aerosols that includes emission of super coarse dust
- The parameterization enables models to reproduce measurements of super coarse atmospheric dust near dust source regions
- The remaining underestimation of super coarse dust over dust outflow regions is likely due to errors in models' deposition processes

## 1 Introduction

Mineral dust is an important component of the Earth system, impacting Earth's radiation budget, clouds, precipitation, biogeochemistry, and air quality (Balkanski et al., 2021; Chen et al., 2019; Guieu et al., 2019; Jickells et al., 2005; Kosmopoulos et al., 2017; Meng et al., 2021). Because all these impacts depend sensitively on dust size (Mahowald et al., 2014), global aerosol models need to accurately represent the dust particle size distribution (PSD) to quantify the various dust impacts on the Earth system. Recent dust PSD measurements have indicated that super coarse (diameter >10 μm) dust particles are more prevalent than previously thought (Ryder et al., 2018, 2019; van der Does et al., 2018; Varga et al., 2021). Indeed, recent model estimates suggest an atmospheric load of ∼10 Tg of super coarse dust, which represents about a quarter of the total atmospheric loading of particulate matter and adds an estimated warming effect of around 0.10 W m^−2^ by absorbing both short‐wave and long‐wave radiation (Adebiyi & Kok, 2020; Di Biagio et al., 2020; Kok et al., 2017, 2021). However, some current models do not include super coarse dust (Checa‐Garcia et al., 2021; Wu et al., 2020; Zhao et al., 2022). Other models that do represent super coarse dust underestimate their abundance by over an order of magnitude (Adebiyi & Kok, 2020). This omission or underestimation of super coarse dust by models hinder our quantitative understanding of how dust aerosols affect the current and future climates (Adebiyi & Kok, 2020; Di Biagio et al., 2020; Ryder et al., 2019).

Because the underestimation of super coarse dust by models could be due to an underestimation of super coarse dust emission, we develop a new parameterization of the PSD of emitted dust that accounts for the emission of super coarse dust (Section 2). We implement this new parameterization in the Community Earth System Model (CESM, version 1.2) and evaluate the simulated atmospheric dust PSD with in situ aircraft measurements (Sections 3 and 4). We find that the CESM with this new parameterization reproduces the abundance of super coarse dust close to dust source regions. We also discuss possible reasons why the model still underestimates super coarse dust further from source regions (Section 5).

## 2 Derivation of Parameterization of Emitted Dust Size Distribution Including Super Coarse Dust

In this section, we first review the original brittle fragmentation theory for parameterizing the emitted dust size distribution (Section 2.1). We then extend it to account for super coarse dust emission (Section 2.2).

### 2.1 Original Brittle Fragmentation Theory

The emitted dust PSD depends on the physical process of dust emission. Instead of being lifted directly by wind, dust aerosols are therefore usually emitted from the energetic impacts of bouncing or saltating larger sand‐sized particles (∼70–500 μm) onto dust aggregates in the soil, a process known as sandblasting (Kok et al., 2014; Shao, 2008). When a saltating particle strikes a soil dust aggregate, the impact creates elastic waves that can rupture the interparticle bonds between particles in the dust aggregate. Since dry dust aggregates are usually brittle (Braunack et al., 1979; Perfect & Kay, 1995), Kok (2011) hypothesized that dry soil dust aggregates shatter upon impact by an energetic saltating particle in much the same way that brittle materials, such as glass, shatter upon a sufficiently energetic impact. This hypothesis is supported by the observation that measurements of the emitted dust size distribution follow the power law observed for brittle fragmentation in the 1–10 μm diameter range (Figure S1 in Supporting Information S1).

By assuming that most dust aerosol emissions are the result of fragmentation of dry soil dust aggregates by impacting saltators, Kok (2011) proposed an expression for the size distribution of emitted dust aerosols with diameter smaller than 20 μm:

(1) $$\frac{dV_{\text{emis}}}{dlnD}\;=\;\frac{D}{c_{v}}\left[1+\text{erf}\left(\frac{\ln(D/\bar{D_{s}})}{\sqrt{2}\ln\;\sigma_{s}}\right)\right]\text{exp}\left[-{\left(\frac{D}{\lambda }\right)}^{3}\right],$$

where $V_{\text{emis}}\;$ is the normalized volume of dust aerosol with geometric diameter D, $c_{v}\;$ is a normalization constant ensuring that the integral over $\frac{dV_{\text{emis}}}{dlnD}$ equals 1, and $\bar{D_{s}}$ = 3.4 μm and $\sigma_{s}$ = 3.0 are the median diameter and geometric standard deviation of the log‐normal distribution of the fully dispersed soil size distribution. The parameter λ is the side crack propagation length, which denotes the propagation distance of side branches of cracks created in the soil dust aggregate by a fragmenting impact. Experimental data indicate that λ is of the order of 10% of the size of the object being fragmented (Herrmann & Roux, 2014; Oddershede et al., 1993). Based on a fit to the available data at that time, Kok (2011) used λ = 12 μm, thereby implicitly assuming that the fragmentation of all soil dust aggregates are governed by a similar side crack propagation length, even though soil dust aggregates can differ notably in size (e.g., Chatenet et al., 1996; Klose et al., 2017).

**Figure 1:**
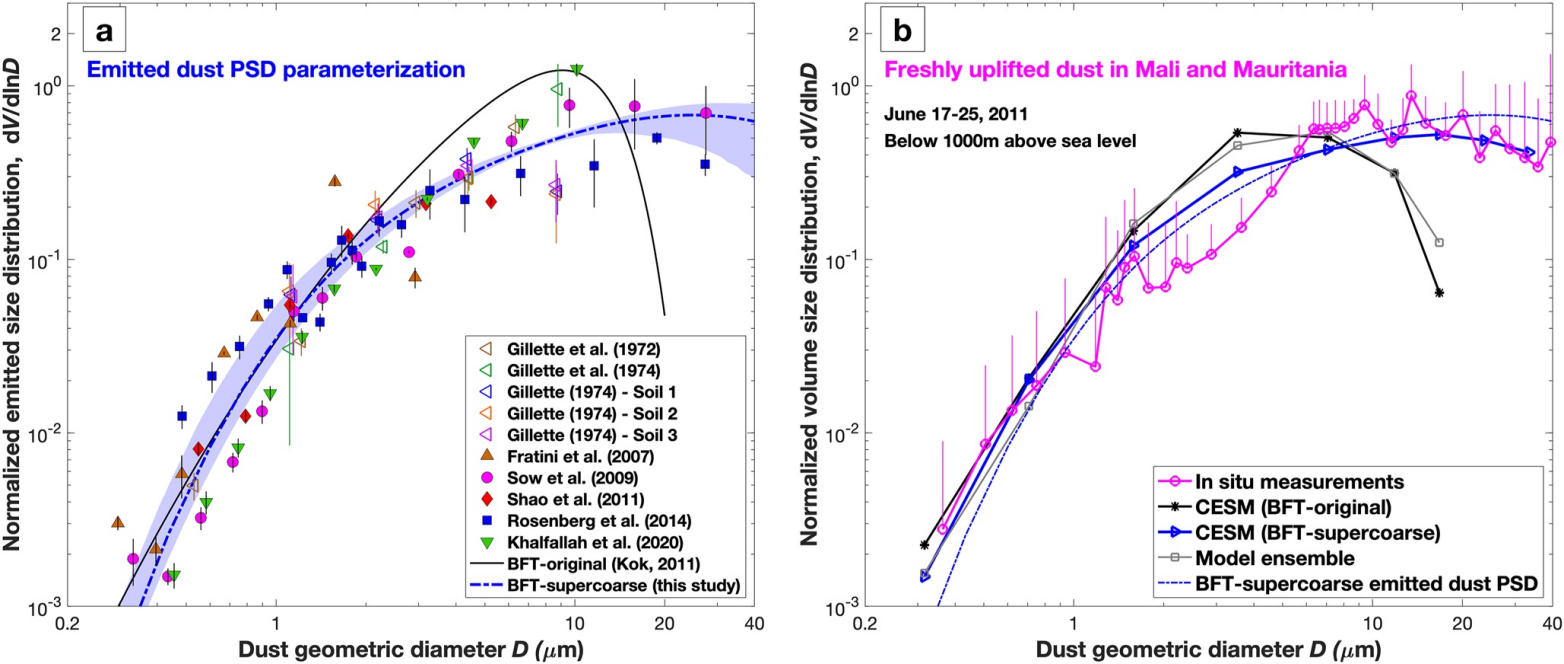
The parameterization for the emission of super coarse dust reproduces measurements of emitted and freshly lifted super coarse dust. Shown are (a) comparison between the parameterization and measurements of the normalized PSD of emitted dust and (b) comparison of the simulated and measured atmospheric PSD of freshly uplifted dust. In (a), different markers denote observations of the emitted dust PSD from different studies, which were described and corrected to geometric diameter as detailed in Huang et al. (2021). Vertical error bars denote the standard error of measurements under various wind events at a given soil (see Kok et al., 2017), the black line denotes the dust PSD predicted by the original brittle fragmentation theory (BFT‐original), and the blue dash‐dotted line denotes the parameterization of emitted dust developed in this study (BFT‐supercoarse). Blue shading denotes the 68% confidence interval. In (b), the magenta open circles denote the average PSD of freshly uplifted dust over Mali and Maritania (∼23.5°N, 7°W) during FENNEC‐2011 campaign (flights B600, B601, B602 and B610 at altitudes below 1,000 m). Vertical error bars indicate one standard deviation of the data; only upward error bars are shown for clarity. The black and blue lines with different markers show the simulated atmospheric dust PSD with the original emitted dust PSD (BFT‐original) and the new emitted dust PSD parameterization (BFT‐supercoarse; the blue dash‐dotted line), respectively. The gray line denotes the seasonally averaged (JJA) dust PSD from an ensemble of global model simulations (Adebiyi et al., 2020). All curves are normalized to yield unity when integrated over the 0.1–20 μm diameter range.

This brittle fragmentation theory (hereafter BFT‐original; Equation 1) is in good agreement with measurements of the emitted dust size distribution for dust particles smaller than 10 μm in diameter (Kok, 2011; Rosenberg et al., 2014; Shao et al., 2011), including several data sets published after the publication of BFT‐original (Figure 1a). Therefore, it has been extensively used in global aerosol models (e.g., Albani et al., 2014; Ito et al., 2012; Zhang et al., 2013). However, recent work has indicated that BFT‐original substantially underestimates the emission of super coarse dust (diameter >10 μm; Huang et al., 2021; Rosenberg et al., 2014). The emission in that size range is largely determined by the value of the side crack propagation length (λ).

### 2.2 Extending Brittle Fragmentation Theory to Account for Super Coarse Dust Emission

We develop a parameterization that accounts for super coarse dust emission (hereafter BFT‐supercoarse). We do so by extending brittle fragmentation theory (Equation 1) to include a more realistic description of the size‐crack propagation length (λ), which determines the cut‐off diameter above which aerosols are no longer created through fragmentation. Since λ scales with the size of the object (e.g., soil dust aggregate) being fragmented (Herrmann & Roux, 2014; Oddershede et al., 1993), it is proportional to the size of the soil dust aggregate being fragmented. That is,

(2) $$\lambda \;=\;f_{\lambda }D_{agg},$$

where $f_{\lambda }$ ∼ 0.15 is the ratio of λ to the dust aggregate size (Herrmann & Roux, 2014; Oddershede et al., 1993) and $D_{\text{agg}}$ is the diameter of the fragmented soil dust aggregate. We account for the dependence of λ on the soil aggregate diameter by substituting Equation 2 into Equation 1, accounting for the probability distribution $P_{\text{agg}}$ ($D_{\text{agg}}$) of the diameter of soil aggregates, and then integrating over the size of soil dust aggregates that can be fragmented to produce dust aerosols:

(3) $$\frac{dV_{\text{emis}}}{dlnD}\;=\;\frac{D}{c_{v}}\left[1+\text{erf}\left(\frac{\ln(D/\bar{D_{s}})}{\sqrt{2}\ln\;\sigma_{s}}\right)\right]\;\overset{\infty }{\underset{0}{\int }}\exp{\left(-\frac{D}{f_{\lambda }D_{\text{agg}}}\right)}^{3}P_{\text{agg}}\left(D_{\text{agg}}\right)dD_{\text{agg}},$$

where $V_{\text{emis}}\;$ is the normalized volume of dust aerosol with size D, $c_{v}\;$ is a normalization constant ensuring that the integral of $\frac{dV_{\text{emis}}}{dlnD}$ equals 1 over the size range 0.1–20 μm (following Kok, 2011), and $\bar{D_{s}}=1.13$ ± 0.58 μm and $\sigma_{s}$ = 1.92 ± 0.25 were obtained by optimizing agreement against measurements, as detailed in Kok et al. (2017) and Huang et al. (2021). Using $P_{\text{agg}}\left(D_{\text{agg}}\right)=\;\delta$ ($D_{agg}$ − λ/$f_{\lambda }$) in Equation 3 recovers Equation 1.

The available measurements indicate that the size distribution of minimally dispersed aggregated soil particles in arid soils generally follows a lognormal distribution (Butler et al., 2013; Chatenet et al., 1996; Klose et al., 2017). Assuming that the size distribution of fragmented soil dust aggregates (*P*_agg_) bears a close resemblance to the size distribution of undisturbed soil particles, we can write $P_{\text{agg}}\left(D_{\text{agg}}\right)$ as:

(4) $$P_{\text{agg}}\left(D_{\text{agg}}\right)=\left(\frac{1}{D_{\text{agg}\;}\;\ln\left(\sigma_{\text{agg}}\right)\sqrt{2\pi }}\right)\text{exp}\left(-\frac{{\left(\ln\left(D_{\text{agg}}\right)\;-\;\ln\left(\bar{D_{\text{agg}}}\right)\right)}^{2}}{{2\left(\ln\left(\sigma_{\text{agg}}\right)\right)}^{2}}\right),$$

where $\sigma_{\text{agg}}$ and $\bar{D_{\text{agg}}}\;$ are the geometric standard deviation and median diameter of the log‐normal distribution of the minimally dispersed soil size distribution. We obtain the parameters ($\sigma_{\text{agg}}$ and $\bar{D_{\text{agg}}}$) of the soil dust aggregates size distribution $P_{\text{agg}}\left(D_{\text{agg}}\right)$ in Equation 4 by analyzing a compilation of measurements of the minimally dispersed size distribution in arid soils and fitting lognormal distributions to the measurements. A detailed description of the compilation of soil aggregate PSD measurements can be found in the Supporting Information S1. The results indicate that the parameters of the soil dust aggregate size distribution are relatively constant and do not correlate with soil texture, which is consistent with previous work (Chatenet et al., 1996; Laurent et al., 2006). Therefore, these parameters can be described by a constant median diameter ($\bar{D_{\text{agg}}}\;$) of 127 ± 47 μm and geometric standard deviation ($\sigma_{\text{agg}}$) of 2.95 ± 1.01 for the soil dust aggregate size distribution. We calculate the error bounds (uncertainty) on the theoretical prediction by using a bootstrap method combined with a maximum likelihood method that propagates errors in $\bar{D_{s}}$, $\sigma_{s}$, $\bar{D_{\text{agg}}}$, and $\sigma_{\text{agg}}$, following the approach in Kok et al. (2017).

The new BFT‐supercoarse parameterization derived here is in good agreement with the available measurements of the emission of super coarse dust (Figure 1a). However, there are few measurements of the emitted dust PSD that extend to the super coarse dust size range because such large particles are usually lost in instrument inlets due to their high inertia (Rosenberg et al., 2014; von der Weiden et al., 2009). In order to better determine whether the new emitted dust PSD parameterization reasonably represents the emission of super coarse dust, in the next section we use global aerosol model simulations to further evaluate the parameterization against aircraft measurements of freshly emitted atmospheric dust. These aircraft measurements were acquired by wing‐mounted probes and did not suffer from inlets restricting measurements of coarse particles (Ryder et al., 2015, 2018).

## 3 Methodology for Evaluating Super Coarse Dust Emission Parameterization Against In Situ Measurements Using CESM Simulations

### 3.1 Aircraft Measurements of Atmospheric Dust PSD

We compiled atmospheric dust PSD measurements from several aircraft measurement campaigns near dust source regions and over dust outflow regions (Figure S2 in Supporting Information S1). We used measurements of freshly uplifted dust and aged dust near dust source regions from the FENNEC 2011 aircraft campaign over western North Africa (Ryder et al., 2015). Specifically, we used measurements of (a) freshly uplifted dust (FENNEC flights B600, B601, B602 and B610), (b) advected aged dust emitted from the Atlas Mountains (flights B605 and B606), and (c) advected aged dust emitted from Mauritania (flights B609, B611, B612, and B613). The source regions for dust sampled during each flight were determined using back‐trajectory analysis (Ryder et al., 2015). All the three categories of measurements assumed dust particles to be spherical, and we corrected them to account for dust asphericity following Huang et al. (2021), which is critical because ignoring dust asphericity causes an overestimate of dust particle size at coarse sizes (Figure S5 in Supporting Information S1). We then averaged over all measurements in each of the three categories.

In addition to the FENNEC, we used aircraft measurements of long‐range transported dust over dust outflow regions, including from the Saharan Mineral Dust Experiment (DARPO) over southern Portugal (Wagner et al., 2009), from the FENNEC‐SAL campaign over the Canary islands (Ryder, Highwood, Lai, et al., 2013), and from the Saharan Aerosol Long‐Range Transport and Aerosol‐Cloud‐Interaction experiment (SALTRACE) over Barbados and Cape Verde (Weinzierl et al., 2017). Because exact specifications of the instrumentation used in these campaigns were not conveniently available, these measurements were not corrected for dust optical properties and asphericity (Huang et al., 2021).

A detailed description of all aircraft measurements used in this study is provided in the Supporting Information S2.

### 3.2 CESM Model, Simulations and Model Ensemble

We used CESM version 1.2 (Hurrell et al., 2013) with the Community Atmosphere Model version 4.0 (CAM4; Neale et al., 2010) as the atmosphere component. This version of the model uses externally mixed particle bins to simulate dust. We added four extra logarithmically spaced coarse bins (10–14, 14–20, 20–28, and 28–40 μm) in addition to the four default bins (0.1–1, 1–2.5, 2.5–5, and 5–10 μm). The dust emission module is a physically based dust emission parameterization derived in Kok et al. (2014). The mass fraction of emitted dust in each model bin, which represents the emitted dust PSD in the model, is calculated from the original (BFT‐original) and the new (BFT‐supercoarse) dust emission PSD parameterization. CESM accounts for wet deposition as well as gravitational settling and turbulent deposition processes (see Section 2.8 in Zender et al., 2003), which are dust size and density dependent. We accounted for the effect of dust asphericity by decreasing the gravitational settling velocity by 13% following Huang et al. (2020). A detailed description of the CESM model is provided in the Supporting Information S3.

We conducted CESM simulations nudged toward MERRA2 dynamics. We used a horizontal resolution of 1.9° × 2.5° and 56 vertical levels and simulated the period 2006–2015, which overlaps with the years that available aircraft measurements were taken. For model comparisons against measurements of freshly lifted dust events, we included dust emission only from the grid box from which the sampled dust was emitted, as determined from backwards trajectory analyses (Ryder et al., 2015). For model comparisons against other measurements, including aged dust and long range transported dust, we included dust emission for all grid boxes in the model. We also conducted a set of simulations with the new BFT‐supercoarse PSD parameterization that use dust aerosol densities of 125, 250, 500, and 1,000 kg m^−3^, which are substantially smaller than the physical density of 2,500 kg m^−3^. The objective of these simulations was to quantify the effect of the missing or erroneously described processes that cause an apparent overestimation of the deposition and underestimation of the long‐range transport of super coarse dust (Ansmann et al., 2017; Weinzierl et al., 2017). We averaged our model simulation during the daytime (10:00–18:00 local time) for the days for which measurements were made. We also tested averaging our model simulation over different temporal resolutions and found that the simulated atmospheric dust PSDs shows limited sensitivity to the averaging period (Figure S3 in Supporting Information S1).

To evaluate the performance of current models in simulating super coarse dust, we also compare our simulated atmospheric dust PSD with the atmospheric dust PSD from an ensemble of model simulations. This model ensemble was obtained in Adebiyi et al. (2020) and used simulations from six different models that provide seasonally averaged atmospheric dust PSD (Supporting Information S3.2).

## 4 Evaluation of Super Coarse Dust Emission Parameterization Against In Situ Measurements Using CESM Simulations

We first evaluate the simulations against in situ aircraft measurements taken close to dust source regions, which shows a significant improvement of the new emitted dust PSD parameterization in reproducing the super coarse atmospheric dust over dust source regions (Section 4.1). Subsequently, we evaluate the simulated dust PSD against measurements taken in dust outflow regions, finding that CESM still underestimates long‐range transported super coarse atmospheric dust (Section 4.2).

### 4.1 Comparison Against Measurements Near Dust Source Regions

Measurements of the atmospheric dust size distribution over dust source regions are well suited to evaluate the accuracy of the super coarse dust PSD parameterization because these measurements are minimally affected by dust transport and deposition, which models struggle to simulate accurately (Adebiyi & Kok, 2020; Di Biagio et al., 2020). We thus compare the atmospheric dust PSD from the simulations with the BFT‐original and BFT‐supercoarse emitted dust PSDs against in situ measurements of the PSD of freshly uplifted dust in western North Africa from the FENNEC 2011 campaign (Figure 1b). As expected, the simulated freshly uplifted atmospheric dust PSD is highly similar to the parameterized emitted dust PSD. We find that the simulation with the new BFT‐supercoarse dust emission PSD parameterization reproduces the measurements of the PSD of freshly lifted atmospheric dust over the dust source region, notably including the super coarse dust size range. In contrast, both the model ensemble and the CESM simulation with the BFT‐original parameterization substantially underestimate the abundance of super coarse atmospheric dust. This result indicates that the new dust emission PSD parameterization can reasonably represent the emission of super coarse dust.

**Figure 2:**
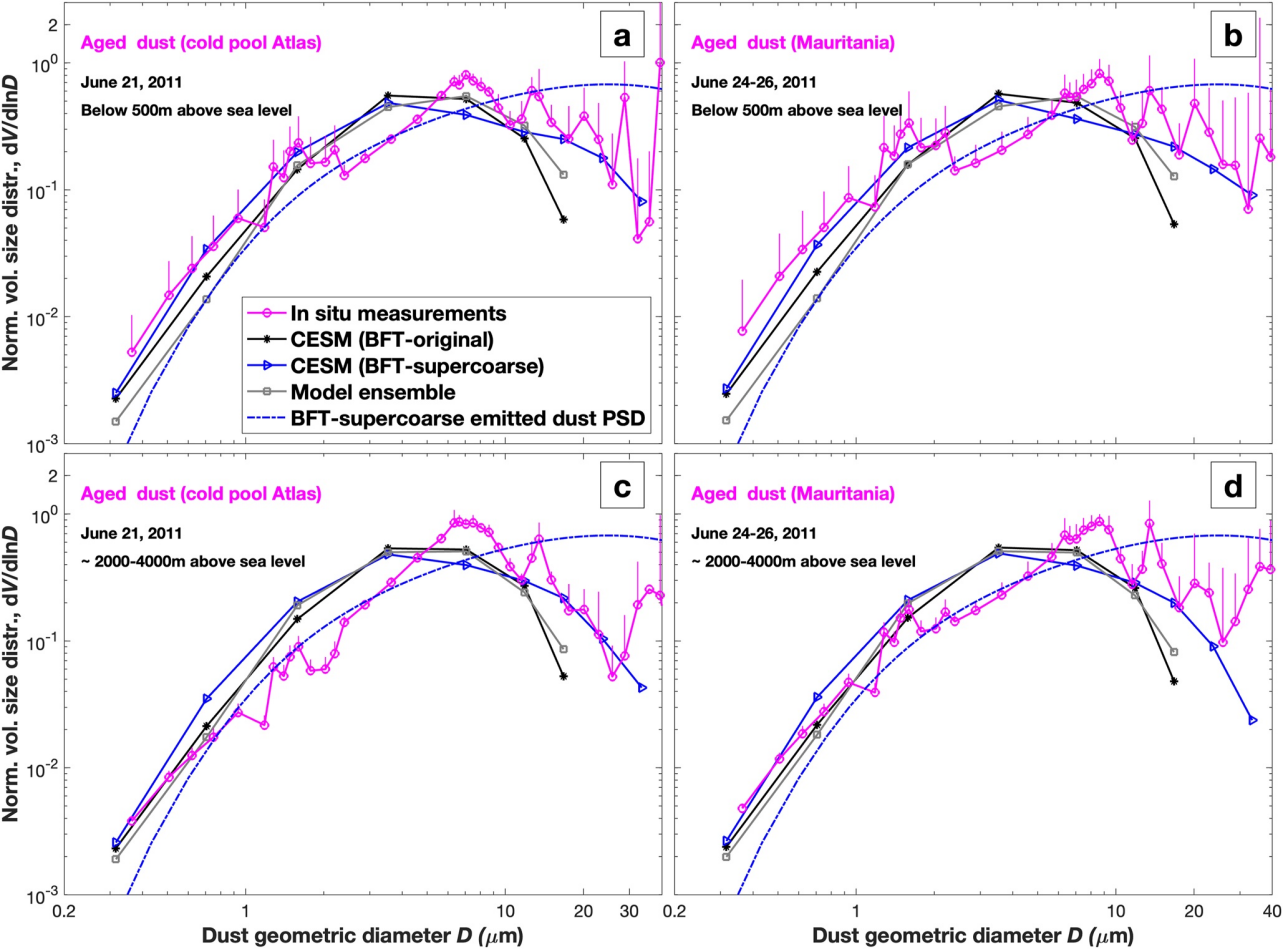
Comparison between simulations and measurements of the PSD of aged dust near source regions. Shown are CESM simulations using both the upgraded BFT‐supercoarse (blue line with triangle markers) and the original BFT‐original dust emission PSD (black line with asterisk markers), as well as the seasonally averaged dust PSD (gray line with square markers) from an ensemble of model simulations (Adebiyi et al., 2020). The blue dash‐dotted line denotes the parameterization of emitted dust developed in this study (BFT‐supercoarse). Atmospheric dust PSD measurements (magenta open circles) are from FENNEC 2011 flights that measured aged dust events, including aged dust from a cold pool near the Atlas mountains (flights B605 and B606) and from Mauritania (flights B609, B611‐613) (Ryder et al., 2015). Vertical error bars indicate one standard deviation of the data; only upward error bars are shown for clarity. All curves are normalized to yield unity when integrated over the 0.1–20 μm diameter range.

Next, we evaluate the model performance with BFT‐supercoarse against aged atmospheric dust PSD measurements taken close to dust source regions. These measurements sampled atmospheric dust that was not locally emitted but rather was aged and transported from nearby dust source regions (Ryder et al., 2015). Figure 2 shows the atmospheric dust PSD simulated using both the BFT‐original and the BFT‐supercoarse emitted dust PSDs, as well as the PSD from the model ensemble. We find that the simulation with the new BFT‐supercoarse parameterization is consistent with measurements of the PSD of aged dust close to the surface (Figures 2a and 2b), with super coarse dust with *D* > 20 μm becoming somewhat underestimated with increasing altitude (Figures 2c and 2d). In contrast, the model ensemble and the simulation with the BFT‐original emitted dust PSD substantially underestimates super coarse dust at all altitudes.

### 4.2 Underestimation of Super Coarse Atmospheric Dust Over Dust Outflow Regions

**Figure 3:**
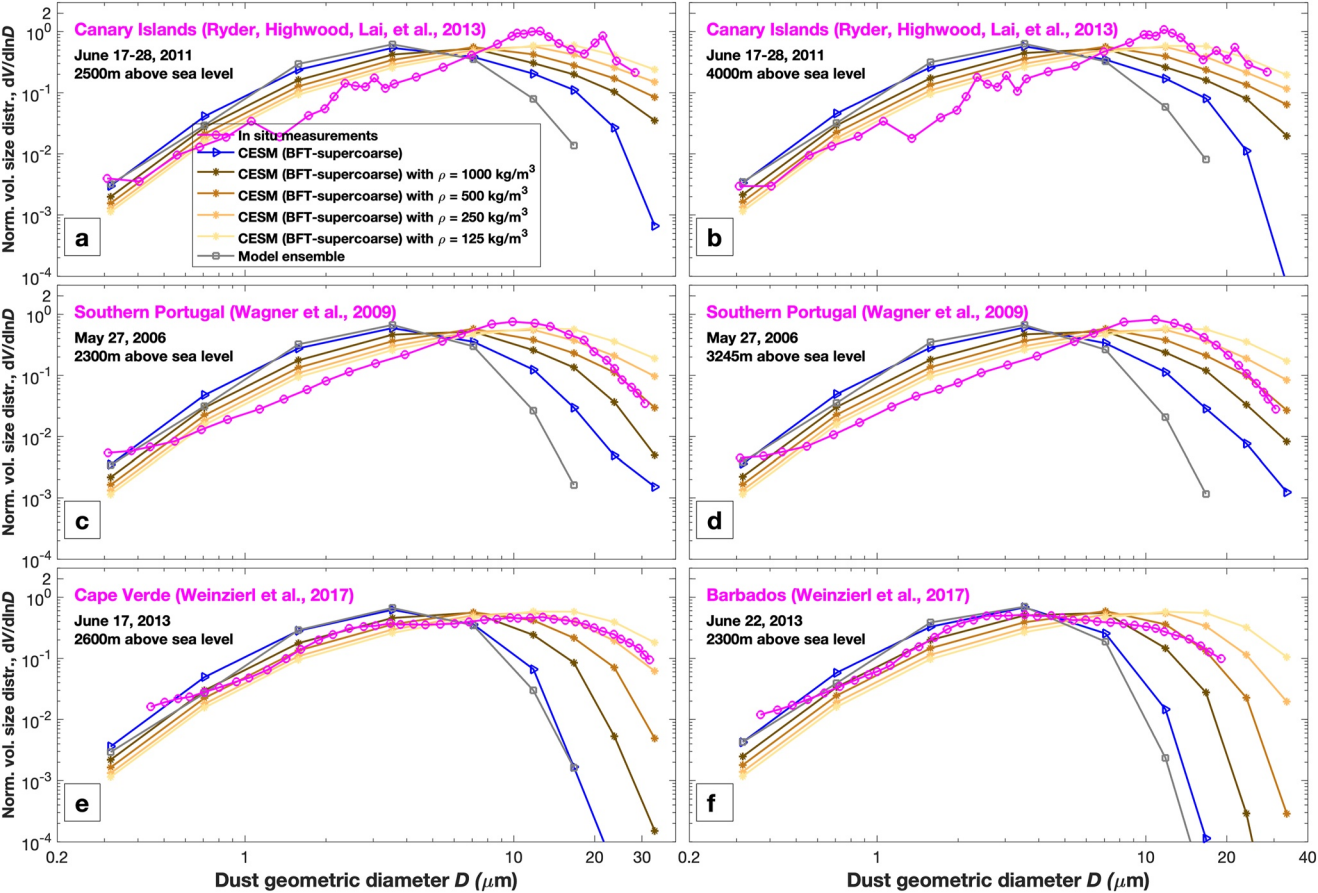
Comparison between simulations and measurements of the dust PSD in dust outflow regions. Simulated atmospheric dust PSDs include a simulation using the default dust aerosol density (2,500 kg m^−3^, blue line with triangle markers) and four simulations using smaller dust aerosol densities (1,000 kg m^−3^, dark brown line; 500 kg m^−3^, brown line; 250 kg m^−3^, orange line; 125 kg m^−3^, light yellow line); all simulations use the BFT‐supercoarse emitted dust PSD. The gray line is the seasonally averaged dust PSD from an ensemble of model simulations (Adebiyi et al., 2020). Measurements (magenta open circles) include the dust PSD over the Canary Islands (∼28°N, 16°W) at two altitudes (2500 and 4000m) (Ryder, Highwood, Lai, et al., 2013), over southern Portugal (∼38°N, 8°W) at two altitudes (2300 and 3245m) (Wagner et al., 2009), and at Cape Verde (∼15°N, 23°W) and Barbados (∼12°N, 60°W) (Weinzierl et al., 2017). All curves are normalized to yield unity when integrated over the 0.1–20 μm diameter range.

To examine whether our new parameterization enables models to also effectively represent super coarse dust further away from dust source regions, we compare the simulated atmospheric dust PSD against measurements taken in dust outflow regions. We find that, despite the significant improvement produced by BFT‐supercoarse in representing super coarse dust near dust source regions (Figures 1b and 2), our CESM simulation with BFT‐supercoarse still underestimates super coarse dust in dust outflow regions, such as the eastern Atlantic Ocean and the Caribbean region (Figure 3). Moreover, the magnitude of the underestimation becomes larger as the distance of the measurement location from dust source regions increases. For example, the underestimation of super coarse dust with *D* > 20 μm over Barbados (Figure 3f) is substantially larger than that over Cape Verde (Figure 3e). This suggests that the underestimation is due to errors in the model's dust deposition processes or vertical numerical diffusion during dust transport.

In order to quantify model errors due to the underestimation of the super coarse dust lifetime, we test whether a decreased dust aerosol density yields a more realistic abundance of super coarse dust further from source regions. Indeed, our sensitivity simulations using smaller dust aerosol densities show greatly improved agreement with observations over the desert outflow regions (Figure 3). Optimal agreement is produced with simulations using 125 and 250 kg/m3, for which the lifetime of super coarse dust is increased by over an order of magnitude (Figure S6 in Supporting Information S1). This suggests that the equivalent effect of possible errors in dust deposition processes during dust transport is to underestimate the lifetime of super coarse dust by an order of magnitude. Although it would be preferable to address the model errors that cause the underestimation of super coarse dust lifetime, our results suggest that decreasing the modeled dust aerosol density by 10–20 times its physical value of ∼2,500 kg/m3 could be useful in more accurately simulating the long‐range transport of super coarse dust and also further improves model agreement against measurements of super coarse dust near source regions (Figure S7 in Supporting Information S1).

## 5 Discussion and Perspectives

We have presented a new parameterization for the emitted dust PSD that accounts for the emission of super coarse dust (Figure 1a). Our evaluation of this new parameterization using CESM has shown that it can reproduce the abundance of super coarse dust close to dust source regions (Figures 1b and 2). However, we also find that CESM still greatly underestimates super coarse dust far from source regions (Figure 3). This work is subject to several important limitations, which are discussed in more detail in the Supporting Information S4.

These findings imply that errors in model processes besides emission, most likely in dust deposition and numerical diffusion, contribute to the underestimation of super coarse dust in models, as previously hypothesized (Adebiyi & Kok, 2020; van der Does et al., 2018). Such possible model errors include, first, an overestimation of the gravitational setting speed of (super) coarse dust. This could be because vertical electric fields generated by charged dust particles in the atmosphere might generate electric forces that counter gravitational settling (Renard et al., 2018; Ulanowski et al., 2007), or because turbulence in dusty air layers counteracts gravitational settling (Gasteiger et al., 2017). Second, the vertical transport of (super) coarse dust might be underestimated. In particular, a recent study has shown that topography greatly enhances the upward vertical transport of super coarse dust in the boundary layer (Heisel et al., 2021). This effect is not fully accounted for in most models and could help explain the remaining slight underestimation of super coarse dust near source regions (Figures 1b and 2). Additionally, the current available meteorological wind field that drives the model might not capture convection events that lift super coarse dust higher into the boundary layer (Cowie et al., 2015; Roberts et al., 2017). Third, although the Piecewise Parabolic Method transport scheme used in CAM4 produces relatively little numerical diffusion (Neale et al., 2010), it is nonetheless possible that numerical diffusion in CAM4 causes an overestimate of dust deposition (Ginoux, 2003; Rastigejev et al., 2010). Therefore, improvements in dry deposition schemes, meteorological input fields, and advective transport schemes might be needed to correctly simulate the long‐range transport of (super) coarse dust. However, until that is achieved, our findings indicate that reducing the dust aerosol density by 10–20 times can serve as a proxy for these missing or erroneously parameterized processes, at least for the CESM model.

## 6 Conclusions

We have extended the brittle fragmentation theory for the emitted dust particle size distribution in order to account for the emission of super coarse dust. This new parameterization could improve current global aerosol models, which generally neglect or greatly underestimate super coarse dust (Adebiyi & Kok, 2020). We find that our parameterization reproduces the abundance of super coarse dust close to dust source regions. However, the CESM model still substantially underestimates super coarse dust in dust outflow regions, presumably due to errors in numerical diffusion or missing processes during dust transport and deposition. We find that the net effect of these model errors and missing processes is to underestimate the super coarse dust lifetime by an order of magnitude, which is equivalent to decreasing the effective dust aerosol density in our model (CESM) to an order of magnitude less than its physical value of ∼2,500 kg/m^3^. These results for the CESM model suggest that the underestimation of super coarse atmospheric dust by models is in part due to the underestimation of the emission of super coarse dust, which can be resolved by implementing the parameterization presented here, and in part due to errors in deposition processes, which requires further work to resolve but might be ameliorated by artificially reducing the dust density.

## Supporting information

Supporting Information S1

## Data Availability

A supplemental file containing the dust mass fractions for dust bins covering the 0.1–100 μm size range is available in this Zenodo repository (https://doi.org/10.5281/zenodo.6344524) with a DOI of 10.5281/zenodo.6344524. The modified CESM source codes are available in this Zenodo repository (https://doi.org/10.5281/zenodo.6344441) with a DOI of 10.5281/zenodo.6344441. The data on which Tables S1 and S2 in Supporting Information S1 are based are available in Chandler et al. (2004), Li et al. (2014), Mei et al. (2004), Swet and Katra (2016), Liu et al. (1998), Su et al. (2007), Klose et al. (2017), Shao et al. (2011), Ryder, Highwood, Rosenberg, et al. (2013) and Ryder et al. (2015). Data in Figure 1a are available in Fratini et al. (2007), Gillette (1974), Gillette et al. (1972, 1974), Khalfallah et al. (2020), and Sow et al. (2009).
