# Supplementary material for: Improved Parameterization for the Size Distribution of Emitted Dust Aerosols Reduces Model Underestimation of Super Coarse Dust
Source: Geophys Res Lett. 2022 Apr 19;49(8):e2021GL097287. doi: 10.1029/2021GL097287 (PMC9286626; doi:10.1029/2021GL097287)
Supplement: Supplementary file 1 — Supporting Information S1 [file GRL-49-0-s001.docx]

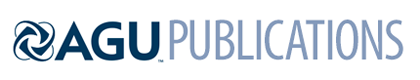


*Geophysical Research Letters*

Supporting Information for

**Improved Parameterization for the Size Distribution of Emitted Dust Aerosols Reduces Model Underestimation of Super Coarse Dust**

Jun Meng^1^, Yue Huang^1,a^, Danny M. Leung^1^, Longlei Li^2^, Adeyemi A. Adebiyi^1,b^, Claire L. Ryder^3^, Natalie M. Mahowald^2^, Jasper F. Kok^1^

^1^Department of Atmospheric and Oceanic Sciences, University of California, Los Angeles, CA 90095, USA

^2^Department of Earth and Atmospheric Sciences, Cornell University, Ithaca, NY 14850, USA

^3^Department of Meteorology, University of Reading, RG6 6BB, Reading UK

^a^Now at Earth Institute, Columbia University, New York, NY 10025, USA and NASA Goddard Institute for Space Studies, New York, NY 10025, USA

^b^Now at Department of Life & Environmental Sciences, University of California, Merced, CA 95343, USA

Corresponding author: Jun Meng (jun.meng@ucla.edu)

**Contents of this file**

- Text S1. Description of the dry aggregate soil size distributions collected from previous studies.
- Text S2. Description of the atmospheric dust size distribution measurements and corrections.
- Text S3. Description of CESM, simulations and the model ensemble used in this study.
- Text S4. Discussion of the limitations of this study.
- Figure S1. Comparison of measurements of the emitted dust size distribution with the brittle fragmentation power law in the 1–10 μm size range.
- Figure S2. Locations of atmospheric PSD measurements campaigns used in this study.
- Figure S3. Simulations showing the sensitivity of the simulated freshly uplifted atmospheric dust PSD to whether dust emissions are included from all grid boxes or just the grid box identified as the dust source and the simulated atmospheric PSD averaged at different temporal resolutions.
- Figure S4. An example of a soil particle size distribution (PSD) for an arid soil in the Tarim Basin from Li et al. (2014).
- Figure S5. Effect of accounting for asphericity on FENNEC PSD measurements for FENNEC flight averaged PSD measurements (flights B600, B601, B602 and B610).
- Figure S6. Simulated atmospheric dust lifetime.
- Figure S7. Comparison between simulations with reduced dust density and measurements of the dust PSD close to dust source regions.
- Tables S1. Dry aggregate size distribution data collected from different studies for this paper.
- Tables S2. Information of each flight in FENNEC 2011 used in this study.

**Text S1. Description of the dry aggregate soil size distributions collected from previous studies**

We searched the literature for studies that obtained undisturbed soil PSDs following two main criteria. First, we only used studies that were conducted in desert dust source regions (mostly arid and some semi-arid regions). Other studies conducted over non-arid regions, such as rainforests and wetlands, have soil PSDs that differ substantially from those of desert soils since moisture and other organics will adhere soil particles into larger aggregates compared with desertic soils (Leung et al., 2020). The differences between arid and non-arid soil PSDs are plotted and discussed in Leung et al. (2020). Here, we define dust emission regions following Hurrell et al. (2013) and Kok, Albani, et al. (2014), that the leaf area index (LAI) must be smaller than or equal to a threshold value ${LAI}_{thr}$. We set ${LAI}_{thr}=1$for our study. The second criterion is that we only select soil PSDs sampled by dry sedimentation techniques (also called dry sieving). Studies usually determine the soil texture of the soil samples by the wet sedimentation technique (or called wet sieving), which involves water or other solutions to break down soil aggregates into disaggregated particles (Chatenet et al., 1996). In contrast, dry sieving causes a minimal disruption to soil aggregates and the resulting soil PSD is thus more representative of the in-situ, aggregated soil PSD (Chatenet et al., 1996; Klose et al., 2017). Using these two criteria, we selected eight studies with a total of 26 soil PSDs from dust source regions that were dry-sieved (see Table S1). The first six studies in Table S1 (Liu et al., 1998; Chandler et al., 2004; Mei et al., 2004; Su et al., 2007; Li et al., 2014; Swet & Katra, 2016) provided the used sieve mesh sizes and the percentages of soil particles that passed through the different sieves, which are listed in Table S1. Some studies used sieve meshes larger than 2000 μm, but here we only account for soil PSDs smaller than 2000 μm as even larger aggregates are likely too large to get fragmented by an impacting saltating particle. Thus, the percentages of the different soil particle sizes listed in Table S1 might not add up to 100%. In addition, the last two papers (Shao et al., 2011; Martina Klose et al., 2017) did not provide mesh sizes and instead provided cumulative PSD plots, from which we extracted the particle size at 10% intervals from their plots. These data are also included in the fourth and fifth columns.

For each soil PSD in our compilation (Table S1), we obtained the median $D_{agg}$ (in μm) and geometric standard deviation (GSD) $\sigma_{agg}$ of the soil aggregate PSDs. Although we can directly use the bin sizes and percentages (Figure S4a) to estimate the median, it will result in a $D_{agg}$ that has the same value as the median bin size, as all soil particles within a bin are assumed to have the same size as the discrete bin size itself. To increase the precision of the median diameter to 1 μm, we first divide the percentages by their corresponding bin width to get the PSD (which transforms Figure S4a to Figure S4b), and then divide the bins into a total of 2000 bins with a width of 1 μm, from 0 μm to 2000 μm (Fig. S4c). We can then identify the 1 μm wide bin that corresponds to the 50^th^ percentile of the PSD to be the median diameter $D_{agg}$. Here we assume the percentages to be constants within each bin, as we do not have any additional information about the sub-bin variability of the percentages from the studies. Figure S4 shows an example of a PSD from Li et al. (2014) over the Tarim Basin for arid soil. Figure S4a shows the percentages of soil particles passing through each sieve opening as a bar plot, which characterizes the probabilities of soil particles residing in each bin. Figure. S4b shows our resulting PSD as $dV/\mathrm{dln}D$ vs $D$ using the given percentages in Figure S4a, in which the sum of the PSD area across each bin interval will be equal to the percentage of the bin in Figure S4a. We then obtain the median and the GSD of the PSDs, which are listed in Table S1.

We investigated whether the variability of $D_{s}$ and $\sigma_{s}$ across different datasets can be explained by other soil properties, such as soil organic carbon content, pH value, and calcite content, but all these variables display an insignificant correlation with $D_{agg}$ and $\sigma_{agg}$ with *p*-value > 0.1 (Figure 3a in Leung et al., 2020). Here, for simplicity, we take the mean of all data, yielding $D_{agg}=127\pm47 \mu m$ and $\sigma_{agg} =2.95\pm1.01 \mu m$ as the input for Eq. 4 in the main text.

**Text S2. Description of the atmospheric dust size distribution measurements and corrections**

**S2.1 Measurements from aircraft campaigns**

In order to evaluate the accuracy of BFT-supercoarse, we collect atmospheric dust particle size distribution (PSD) measurements from several aircraft campaigns, including the FENNEC project in 2011, the Saharan Aerosol Long-Range Transport and Aerosol–Cloud-Interaction Experi­ment (SALTRACE) in 2013, and the Desert Aerosols over Portugal (DARPO) in 2006. The geographic locations of measurements used here from those campaigns are shown in Figure S2.

**S2.1.1 FENNEC 2011**

The Fennec 2011 aircraft campaign measured dust PSDs in western North Africa during June 2011. A suite of instruments were used to measure the atmospheric dust PSD (see table 3 in Ryder, Highwood, Rosenberg, et al. (2013)) on the FAAM Bae146 atmospheric research aircraft, including a wing-mounted Passive Cavity Aerosol Spectrometer Probe 100X (PCASP), a Cloud Droplet Probe (CDP), and a Cloud Imaging Probe (CIP15). The PCASP measurements covered a nominal diameter range of 0.1 – 3.0 um and were taken at a wavelength of 632.8 nm and with a scattering angle from 35 to 145 degrees. The CDP measurements covered a nominal diameter range of 3 - 50 um and were taken at a wavelength of 658 nm and with a scattering angle from 4-12 degrees. Both PCASP and CDP are optical particle counters (OPCs), and OPCs are by default calibrated against the polystyrene latex spheres (PSLs, m=1.59-0i) (ISO, 2009). Thus, the measurements from PCASP and CDP were in terms of the optical diameter of PSLs and were converted to geometric diameter of spherical dust aerosols with a refractive index (m=1.53-0.001i, Ryder, Highwood, Rosenberg, et al., 2013). We used measurements from PCASP and CDP in this study. Details of the calibration and correction performed on each instrument can be found in Rosenberg et al. (2012) and Ryder, Highwood, Rosenberg, et al. (2013). CIP15 data were not used in this study, since only two diameter data points overlap with the size range evaluated here. Nevertheless, the CIP15 is a valuable constraint when evaluating size distributions extending above 15 µm diameter, particularly since it does not rely on an assumption of refractive index.

We used the dust PSD measurements per flight for the 11 flights shown in Table S2. We focused on the comparison with the horizontal ‘runs’ in the different event categories described in Ryder, Highwood, Rosenberg, et al., (2013) and Ryder et al. (2015), including freshly uplift over Northern Mali (flights b600, b601 and b602) and Mauritania (b610), aged dust from a cold pool near the Atlas mountains (flights b605 and b606), and aged dust over Mauritania (flights b609, b611, b612 and b613). Flights b600, b601 and b602 on June 17-18 were designed to investigate very strong low-level winds over northern Mali, during which some of the largest particles were measured. Flights b605 and b606 on June 21 aimed to sample aged dust that was emitted by a convective system over the Atlas Mountains on the preceding day and propagated southwards over Mauritania. Flight b610 on June 24 sampled dust that was freshly uplifted by the low-level jet over eastern Mauritania. Flights b609, b611, b612 and b613 between June 24 and June 26 sampled aged dust over Mauritania under clear sky and low dust loading conditions. The detailed description of each flight can be found in Ryder et al. (2015). In our study, we combined measurements from the flights that sampled freshly uplifted dust (b600, b601, b602 and b610) and computed a mean PSD to represent freshly emitted dust. We also combined measurements from flights b605 and b606 to represent aged dust from cold pool Atlas and we combined measurements from flights b609, b611, b612 and b613 to represent aged dust over Mauritania. We used the pressure height as the altitude variable for these aircraft data. We averaged the PSD measurements from flights that were grouped in the same category, and then we normalized this PSD over the 0.1-20 μm diameter range (see main text).

We also used dust PSD measurements over the Canary Islands (Ryder, Highwood, Lai, et al., 2013) measured during the FENNEC campaign. The instruments used for these measurements were the same as described previously. The dataset used here consists of 21 vertical profiles of PSD measurements gridded to a 50 m vertical grid resolution. We used data at the altitudes of 2000 m and 4000 m above sea level (ASL) to represent the dust PSD transported in the Saharan Air Layer (SAL).

**S2.1.2 SALTRACE**

SALTRACE (<http://www.pa.op.dlr.de/saltrace/>) was conducted between spring 2013 and summer 2014 across the Atlantic Ocean into the Caribbean. Atmospheric PSD measurements were made on board the German Aerospace Center (DLR) research aircraft Falcon equipped with a suite of in situ instruments, including a Passive Cavity Aerosol Spectrometer Probe (PCASP-100X), a Forward Scattering Spectrometer Probe (FSSP-100), and a Cloud and Aerosol Spectrometer with Depolarization (CAS-DPOL). The details about the instruments can be found in the supplemental material of Weinzierl et al. (2017).

We extracted the dust PSD observations from Figure 9 of Weinzierl et al. (2017) for Cabo Verde on 17 June 2013 and for Barbados on 22 June 2013. The measurement for Cabo Verde was taken at the altitude of 2.6 km and the measurement for Barbados was taken at the altitude of 2.3 km.

**S2.1.3 DARPO**

The Desert Aerosols over Portugal (DARPO) project measured the dust PSD over southern Portugal in May/June 2006. We extracted the dust PSD from Figure 9 of Wagner et al. (2009). Those measurements were taken by the aircraft Falcon flying several legs over Evora at two different altitudes on 27 May 2006. The aircraft was equipped with various particle in situ measuring instruments that covered the aerosol size distribution between 4 nm and 100μm. Details about instrumentation can be found in Weinzierl et al. (2009).

**S2.2 Corrections for the PSD measurements**

As described in the previous section, most of the aircraft PSD measurements are from optical particle counters (OPCs), which are the most widely used sensors to measure the size distributions of dust aerosols in field campaigns (Rosenberg et al., 2012). OPCs determine the size of individual sampled particles based on the scattering light intensity. Manufacturers in general calibrate the relationship between the particle size and the scattering light intensity against polystyrene latex spheres (PSLs) following the international standard ISO 21501-1:2009 (ISO, 2009). This default relationship is problematic for sampled particles that are not PSLs, such as dust aerosols. Since dust aerosols have different particle shapes and refractive indices, and therefore different scattering intensities relative to PSLs, the OPCs need to be calibrated to account for the differences in shape and refractive index between dust aerosols and PSLs.

**S2.2.1 Six key steps in bin correction during the instrument’s calibration**

Huang et al. (2021) used six key steps to calibrate OPCs such that they can accurately determine dust size distributions.

1. Lorenz-Mie theory is used to calculate the scattering cross sections as functions of diameter ($D_{psl}$) and wavelength ($\lambda$) for spherical PSLs (with a well-calibrated refractive index of $1.59-0i$; ISO, 2009), $C_{sca}\left( D_{psl},\lambda\right)$;
2. Lorenz-Mie theory is used to calculate the scattering cross sections as functions of diameter ($D_{sph}$), wavelength ($\lambda$), and refractive index ($m=n-ki$) for spherical dust aerosols, $C_{sca}\left( D_{sph},\lambda,n,k \right)$;
3. the extensive single-scattering database (Meng et al., 2010; Huang et al., 2021) is used to calculate the scattering cross sections as functions of volume-equivalent diameter ($D_{asp}$), wavelength ($\lambda$), and refractive index ($m=n-ki$) for ellipsoidal dust aerosols, $C_{sca}\left( D_{asp},\lambda,n,k \right)$;
4. determining the wavelength- and the dust refractive index-resolved relationship between the diameter of PSLs ($D_{psl}$), the diameter of spherical dust ($D_{sph}$), and the volume-equivalent of ellipsoidal dust ($D_{asp}$) that would generate the same scattering cross section;
5. (a). correcting the dust number size distribution in terms of diameter of PSLs to the number size distribution in terms of diameter of spherical dust as $\frac{dN_{i}}{dln\left( D_{sph,i} \right)}=\frac{dN_{i}}{dln\left( D_{psl,i} \right)}\times\frac{ln\left( D_{psl,i,up} \right)-ln\left( D_{psl,i,dn} \right)}{ln\left( D_{sph,i,up} \right)-ln\left( D_{sph,i,dn} \right)}$, where $i$ is the $i^{\mathrm{th}}$ size bin and $D_{\sim,i,up}$ and $D_{\sim,i,dn}$ are respectively the upper and lower boundaries of the $i^{\mathrm{th}}$ bin;

(b). correcting the dust number size distribution in terms of diameter of PSLs to in terms of volume-equivalent diameter of ellipsoidal dust as $\frac{dN_{i}}{dln\left( D_{asp,i} \right)}=\frac{dN_{i}}{dln\left( D_{psl,i} \right)}\times\frac{ln\left( D_{psl,i,up} \right)-ln\left( D_{psl,i,dn} \right)}{ln\left( D_{aph,i,up} \right)-ln\left( D_{aph,i,dn} \right)}$, where $i$ is the $i^{\mathrm{th}}$ size bin and $D_{\sim,i,up}$ and $D_{\sim,i,dn}$ are respectively the upper and lower boundaries of the $i^{\mathrm{th}}$ bin;

1. Using a polynomial regression on the obtained number size distributions (from step 5a or 5b) to remove and smooth unrealistic wiggles.

In the six steps above, steps 1 and 2 correct OPC size bins accounting for only the difference in the refractive index between PSLs and dust aerosols, steps 2 and 3 correct OPC size bins accounting for only the difference in the particle shape between spherical and ellipsoidal dust, and steps 1, 2, and 3 correct OPC size bins accounting for the differences in both the refractive index and the particle shape.

**S2.2.2 Correction for PSD measurements in this study considering dust asphericity**

The PSD measurements bins from FENNEC 2011 campaign (Ryder, Highwood, Rosenberg, et al., 2013; Ryder, Highwood, Lai, et al., 2013) have been corrected using realistic dust optical properties (m=1.53-0.001i) following Rosenberg et al. (2012), which went through the steps 1, 2, and 5a as described in section S2.2.1. Although Rosenberg et al. (2012) did not account for the effect of dust asphericity in their bin correction process, they rigorously accounted for the uncertainty due to the wiggle pattern of the Lorenz-Mie theory. Specifically, instead of calculating the one-to-one relationship between $D_{psl}$ and $C_{sca}$ at a high diameter resolution (e.g., 3000 diameters logarithmically in between 0.1 and 50 $\mu m$) (as in Huang et al., 2021), they calculated the probability function of the scattering cross section, $f\left( C_{sca} \right)$, for a given size bin of PSLs within a small diameter interval $D_{psl}\pm\Delta D_{psl}$. Similarly, they calculated the probability function of the scattering cross section, $f\left( C_{sca} \right)$, for a given size bin of spherical dust within a small diameter interval $D_{sph}\pm\Delta D_{sph}$. They then linked the diameter of PSLs ($D_{psl}$) and the diameter of spherical dust ($D_{sph}$) that would generate a similar probability function of the scattering cross section. As such, Rosenberg et al. (2012) propagated the uncertainty in the scattering cross section due to the wiggle pattern of the Lorenz-Mie theory into the corrected size bins.

We accounted for the differences in both the refractive index and the particle shape in the bin correction, namely by combining steps 1 and 2 as in Rosenberg et al. (2012) and step 3 as in Huang et al. (2021). Since the flight specific measurements from FENNEC 2011 already corrected for dust refractive index, we correct those data only for dust asphericity following Huang et al. (2021). This is feasible because the scattering cross section calculated by the extensive single-scattering database of ellipsoidal dust (Meng et al., 2010) increases monotonically with the volume-equivalent diameter (see Figs. S2b and S3b of Huang et al. (2021). This smooth pattern (without wiggles as in the Lorenz-Mie results) occurs because we integrated the shape-resolved single-scattering properties of ellipsoidal dust against the globally representative shape distributions of dust aerosols (Huang et al., 2021). By combining steps 1 and 2 as in Rosenberg et al. (2012) and step 3 as in Huang et al. (2021), we accounted for the differences in both the refractive index and the particle shape in the bin correction, as well as the uncertainty due to the non-monotonic nature of Lorenz-Mie theory.

Figure S5 shows that the correction for dust asphericity shifts the FENNEC PSD measurements shifted towards finer particles bins and smaller volume size distribution. These results are consistent with the findings in Huang et al. (2021).

Although we converted FENNEC campaign PSD measurements in terms of optical diameter to geometric diameter following Huang et al. (2021), we were unable to convert the measurements from other flight campaigns (Wagner et al., 2009; Weinzierl et al., 2017) because the required optical parameter information for the measurement instruments was not conveniently available.

**Text S3. Description of CESM, simulations and model ensemble used in this study**

**S3.1 CESM model description**

The Community Earth System Model (CESM) is a fully coupled, global climate model with atmosphere, land, ocean, and sea ice components that can be used to simulate past, present and future climate. This study uses CESM version 1.2.2.1 (Hurrell et al., 2013) with the Community Atmosphere Model version 4.0 (CAM4) (Neale et al., 2010) as the atmosphere component and the Community Land Model version 4.0 (CLM4) (Lawrence et al., 2011) as the land component. This version of the model uses the bin approach and is not able to calculate aerosol-cloud interactions. The dust emission module in CLM4, which calculates the vertical dust emission flux, is a physically based dust emission parameterization derived in Kok, Mahowald, et al. (2014). This vertical dust emission flux is passed to CAM4 to calculate the three-dimensional transport and deposition of dust.

In this study, we ran CAM4 driven by the meteorological fields of the Modern-Era Retrospective Analysis for Research and Applications, version 2 (MERRA-2), which is an atmospheric reanalysis of the modern satellite era produced by NASA’s Global Modeling and Assimilation Office (GMAO) (Gelaro et al., 2017). CAM4 simulated dry and wet dust deposition. Dust dry deposition includes gravitational settling and turbulent deposition processes (Zender et al., 2003). The default dust grain density is set to 2,500 kg m^-3^. Dust wet deposition consists of scavenging from both convective and large scale precipitation simulated in CAM4 (N. M. Mahowald et al., 2006; Neale et al., 2010). Both dry and wet deposition parameterization for dust are size-dependent.

The dust size distribution in CAM4 is implemented in a Bulk Aerosol Model (BAM) parameterization (Neale et al., 2010). By default, CAM4 BAM partitions dust emission fluxes into four size bins, with bin 1 (0.1–1.0 µm), bin 2 (1.0 – 2.5 µm), bin 3 (2.5 – 5.0 µm) and bin 4 (5.0 – 10.0 µm) in diameters (N. M. Mahowald et al., 2006). The fraction distributed into each bin follows the brittle fragmentation theory (BFT) dust size distribution parameterization derived in Kok (2011) (hereafter BFT-original). In this study, we extend the size range of the dust size distribution in CAM4 BAM by adding four extra bins, which are bin 5 (10.0 – 14.0 µm), bin 6 (14.0 – 20.0 µm), bin 7 (20.0 – 28.0 µm) and bin 8 (28.0 – 40.0 µm) in diameters, to represent super coarse dust (D > 10 μm). The dust mass fraction distributed into each bin follows the coarse dust size distribution derived in section 2 in this study (hereafter BFT-supercoarse). The mass fractions for the eight bins in this study are 0.8%, 5.9%, 12.0%, 20.7%, 13.2%, 15.7%, 15.6% and 16.0%, respectively. We also provided a supplemental file containing the dust mass fractions for dust bins with bin interval of 0.1 µm covering 0.1 µm to 40 µm size range, so that future research can easily obtain mass fractions for their particular bin boundaries.

**S3.2 Simulations in this study**

We conducted several simulations using the model described above using a horizontal resolution of 1.9°x2.5° with 56 vertical levels in the years that available observations were taken (i.e., 2006 - 2015). The first simulation uses the original BFT-original dust size distribution parameterization, while the second simulation uses the new BFT-supercoarse dust size distribution parameterization. We also perform separate simulations with BFT-original and BFT-coarse to compare against measurements of freshly lifted dust. These simulations include dust emissions only in the grid box from which the sampled dust originated according to several methods applied to determine dust sources (Ryder et al., 2015). The objective of these simulations is to evaluate whether BFT-supercoarse can adequately represent the super coarse dust contribution to freshly lifted dust, which is most representative of emitted dust (Figure S3). For model comparisons against measurements of freshly lifted dust events, we included dust emission only from the grid box from which the sampled dust was emitted, as determined from backwards trajectory analyses (Ryder et al., 2015). For model comparisons against other measurements, including aged dust and long range transported dust, we included dust emission for all grid boxes in the model. We averaged our model simulation during the daytime (10:00 -18:00 local time) for the days for which measurements were made. We also tested averaging our model simulation over different temporal resolutions, for example the entire day, the entire month, and the entire season in which the measurement was taken, and found that the timescale mismatch between simulation and measurement has only a limited effect (Figure S3).

We also conduct a set of sensitivity simulations with the new BFT-supercoarse dust size distribution parameterization using a series of smaller dust aerosol densities, namely 125, 250, 500, and 1000 kg m^-3^. The objective of these simulations is to identify the factor by which dust density needs to be reduced to counteract the underestimation of super coarse dust lifetime in simulations of long-range transported super coarse dust.

The model ensemble was obtained in Adebiyi et al. (2020) and includes simulations using the following models: the Goddard Institute for Space Studies (GISS) ModelE, the Weather Research and Forecasting model coupled with Chemistry (WRF-Chem), the Community Earth System Model (CESM), the Goddard Earth Observing System coupled with Chemistry (GEOS-Chem), the ARPEGE-Climate model, and the Integrated Massively Parallel Atmospheric Chemical Transport (IMPACT). The model ensemble provides seasonally averaged atmospheric dust PSD. This is a longer temporal resolution than used for our CESM simulations, but we found that this timescale mismatch has a relatively small impact on our results (Figure S3). WRF-Chem, GEOS-Chem, IMPACT, ARPEGE-Climate model, and CESM used the BFT-original PSD parameterization.

Since the dust volume size distribution increases strongly in the first model bin (diameter 0.1–1.0 µm), we split it for our CESM model simulations and model ensemble into two sub-bins, one with diameter range 0.2 - 0.5 and the other with diameter range 0.5-1 µm. We do so by partitioning the total dust mass in the first bin of our CESM model simulations and model ensemble based on the mass fraction each sub-bin accounts for, which is calculated from the corresponding emitted dust PSD. We used BFT-original for splitting the model ensemble because most of its member models used the BFT-original as the emission PSD. We can do this process offline because transport and deposition processes are treated as size-independent for dust smaller than 1 µm in most models, including CESM. This is also why some models transport all fine dust with diameter < 1 µm in one bin and only split it up into multiple sub-bins for optical calculations (Zender et al., 2003).

**Text S4. Discussion of the limitations in this study**

This work has a few important limitations. First, the emission of super coarse dust depends on the size distribution of soil aggregates, which is uncertain and described here as globally constant due to lack of data (e.g., Shao, 2011; Klose et al., 2017). Second, the derived updated emitted dust PSD inherently assumes that fragmentation of soil aggregates dominates the dust emission process (Kok, 2011a). As such, we do not account for the effect of other emission processes, such as removal of clay coatings, aerodynamic entrainment, and direct emission of soil particles, all of which might dominate for certain soils and environmental conditions (Huang et al., 2019; Klose & Shao, 2012). Moreover, we do not account for a possible dependence of the emitted dust PSD on wind speed (Kok, 2011b; Shao et al., 2020). Third, although we converted FENNEC campaign PSD measurements in terms of optical diameter to geometric diameter following Huang et al. (2021), we were unable to convert the measurements from other flight campaigns (Wagner et al., 2009; Weinzierl et al., 2017) because the required optical parameter information for the measurement instruments was not conveniently available. Based on Huang et al. (2021), accounting for dust asphericity would shift the measured PSD leftwards and downwards in the coarse and super coarse size range. Therefore, we expect that a smaller decrease of dust density would be needed to match these measurements in the sensitivity simulations that apply a dust density reduction. Fourth, a finer resolution model could improve the super dust simulation by resolving more detailed topography (Heisel et al., 2021). Lastly, the model somewhat overestimates dust with diameter below 5 µm relative to the measurements of Ryder et al. (2015). Although the exact cause of this underestimation is unclear, the comparison between measurements and the model results is sensitive to the normalization. Because we focus on super coarse dust simulation, we normalize our results for the size range of 0.1 to 20 µm diameter. However, the overestimation of the <5 µm fraction would be greatly reduced or disappear if the normalization interval was 1-5 µm (e.g., figure 5a in Mahowald et al. (2014)). Another possible reason for this overestimation is that it depends on the experimental study since no such underestimation occurs relative to the measurements of Weinzierl et al. (2017). This overestimation of dust with D < 5 µm could affect the radiative effect of dust (Kok et al., 2017, 2021; Di Biagio et al., 2020).

**Supplementary tables and figures**

**Table S1.** Dry aggregate size distribution data collected from different studies for this paper.

| Study name | **Study region** | **Site name / number / Land or soil type** | **Sieve mesh sizes (μm, we only include sizes up to 2000 μm)** | **% of soil mass passing through sieve (we include data up to 2000 μm, so values do not add up to 100 %)** | **Estimated median diameter (μm)** | **Estimated**  **geometric standard deviation (GSD) diameter** |
| --- | --- | --- | --- | --- | --- | --- |
| Chandler et al. (2004) | Columbia Plateau, WA, USA | No. 19 | 10,30,45,63,90,125  180,250,355,500,  710,1000,1400,2000 | 0.1,5.9,9,11,  4,3,2,2,2,3,  3,3,2,2 | 63 | 3.19 |
|  |  | No. 22 |  | 0.2,6.8,12,8,  11,4,4,2,5,4,  5,4,3,5 | 88 | 3.18 |
|  |  | No. 26 |  | 0.2,7.8,15,16,  19,16,10,3,3,0,  1,0,0,0 | 72 | 2.06 |
| Li et al. (2014) | Tarim Basin, Xinjiang, China | Desert | 50,75,100,250,500,  840,2000 | 4.18,27.24,  22.19,20.63,  3.81,2.15,3.14 | 87 | 2.11 |
|  |  | Farmland |  | 4.33,14.4,8.61,  13.6,4.23,5.93,  7.23 | 120 | 2.78 |
|  |  | Forest |  | 5.68,28.03,  10.13,6.22,2.39,  5.54,6.75 | 74 | 2.67 |
|  |  | Shrubland |  | 2.42,15.57,  13.29,6.26,2.82,  6.12,8.62 | 93 | 2.67 |
| Mei et al. (2004) | Northern China | Silt loam, Yadan landform | 20,50,70,90,110,  180,350,620,1000,  1500 | 6,5,4,8,6,10,36,  14,6,7 | 237 | 3.06 |
|  |  | Gravelly sand, Yinshan |  | 5,10,10,16,6,10,  17,7,6,6 | 109 | 3.06 |
|  |  | Gravelly and sandy loam, Yinshan |  | 5,8,7,11,5,9,27,  10,6,8 | 199 | 3.10 |
|  |  | Gravelly loam, Yinshan |  | 5,5,4,11,6,10,  28,11,7,7 | 217 | 3.05 |
| Swet and Katra (2016) | Negev Desert, Israel | Long-term natural | 63,125,250,500,  1000,2000 | 10.03,21.44,  10.68,8.05,  6.23,8.63 | 138 | 3.02 |
|  |  | Long-term grazing |  | 8.9,36.15,20.52,  8.97,6.45,6.93 | 124 | 2.54 |
| Liu et al. (1998) | Northern China | Gulang, Lanzhou | 50,100,250,500,  1000,2000 | 42.77,32.54,  17.28,4.44,  2.01,0.46 | 60 | 3.22 |
|  |  | Inner Mongolian Plateau |  | 16.35,20.23,  35.91,18.95,  6.52,1.9 | 156 | 3.08 |
|  |  | Tengger Desert |  | 7.63,13.52,  70.63,5.35,  1.72,0.85 | 161 | 2.15 |
| Su et al. (2007) | Hexi Corridor, China | Ari-Sandic Primosol | 250,500,1000,  2000 | 65.2,6.4,5.1,3.7 | 155 | 2.94 |
|  |  | Ustic Cambosol |  | 80.9,6.3,5.4,3.5 | 149 | 2.91 |
|  |  | Siltigi-Otrthic Anthrsol |  | 86.2,8.9,5.0,2.9 | 150 | 2.89 |
|  |  | Calci-Orthic Aridosols |  | 94.6,3.6,4.0,3.0 | 139 | 2.82 |
| Klose et al. (2017) | Lordsburg, NM, USA | A (before vacuuming) | 2,2.4,6,17,50,  90,140,190,240,420 | 10,10,10,10,10,  10,10,10,10,10 | 65 | 6.97 |
|  |  | B (before vacuuming) | 2.6,7,30,85,110,130,  160,190,240,360 | 10,10,10,10,10,  10,10,10,10,10 | 113 | 5.09 |
|  |  | C (before vacuuming) | 100,115,135,145,160,  175,180,200,220,300 | 10,10,10,10,10,  10,10,10,10,10 | 160 | 1.97 |
|  |  | E (before vacuuming) | 50,90,110,120,130,  150,175,200,230,300 | 10,10,10,10,10,  10,10,10,10,10 | 130 | 2.27 |
|  |  | F (before vacuuming) | 22,55,90,110,130,140,  150,170,205,250 | 10,10,10,10,10,  10,10,10,10,10 | 130 | 2.68 |
| Shao et al. (2011) | Murray–Darling River Basin, Australia | Jade (Method A) | 15,25,40,60,88,150,  250,350,450,550,650 | 3.8, 3.8,7.2,9.6,  13.5,23.9,25,  9.6,2.4,0.96,0.16 | 119 | 2.45 |
|  |  |  |  |  |  |  |
| Arithmetic mean of All data (± indicate 1 sD) |  |  |  |  | 127±47 | 2.95±1.01 |

**Table S2**. Information of each flight from FENNEC 2011 campaign used in this study (Ryder, Highwood, Rosenberg, et al., (2013) and Ryder et al., (2015)).

| **Flight Number** | **Date** | **Time, UTC** | **Category** |
| --- | --- | --- | --- |
| b600 | 17 June 2011 | 10:00 – 11:15 | Freshly uplift from Mali |
| b601 | 17 June 2011 | 17:15 – 18:15 | Freshly uplift from Mali |
| b602 | 18 June 2011 | 10:15 – 11:30 | Freshly uplift from Mali |
| b604 | 20 June 2011 | 15:15 – 15:45 | Aged dust (Mauritania) |
| b605 | 21 June 2011 | 10:00 -10:30 | Aged dust (cold pool Atlas) |
| b606 | 21 June 2011 | 16:00 -18:00 | Aged dust (cold pool Atlas) |
| b609 | 24 June 2011 | 13:30 -14:40 | Aged dust (Mauritania) |
| b610 | 25 June 2011 | 9:15 -10:45 | Freshly uplift over Mauritania |
| b611 | 25 June 2011 | 16:30 -10:30 | Aged dust (Mauritania) |
| b612 | 26 June 2011 | 9:30 -10:45 | Aged dust (Mauritania) |
| b613 | 26 June 2011 | 15:45 -18:00 | Aged dust (Mauritania) |


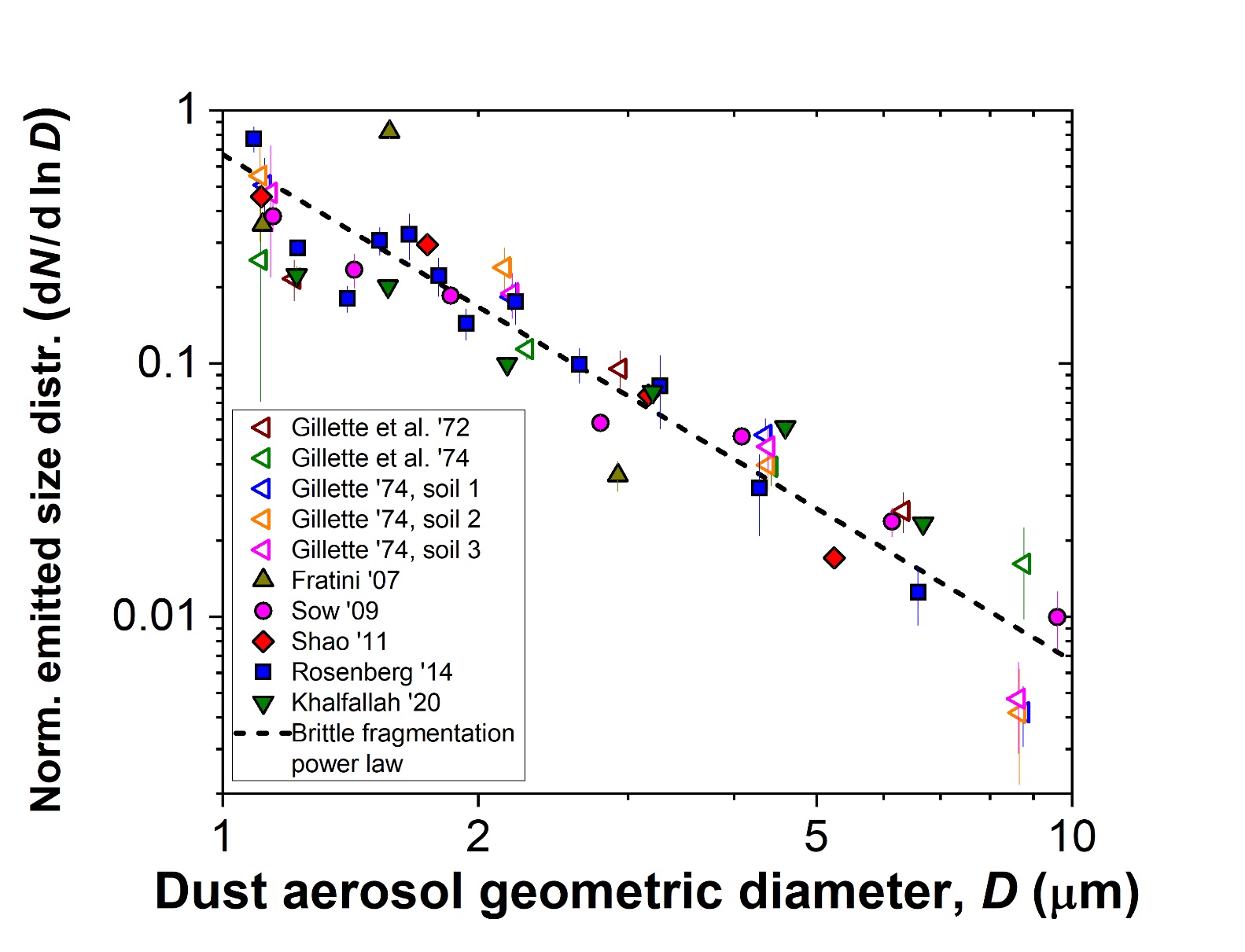


**Figure S1. Comparison of measurements of the emitted dust size distribution with the brittle fragmentation power law in the 1–10 μm size range.** Measurements of the emitted dust size distribution were processed, normalized, and corrected to geometric diameter as detailed in Kok (2011a) and Huang et al. (2021). The dashed line denotes $dN/d\ln D \propto D^{-2}$, which is the power law observed in the scale-invariant fragmentation of brittle materials (Astrom, 2006; Gilvarry and Bergstrom, 1961).


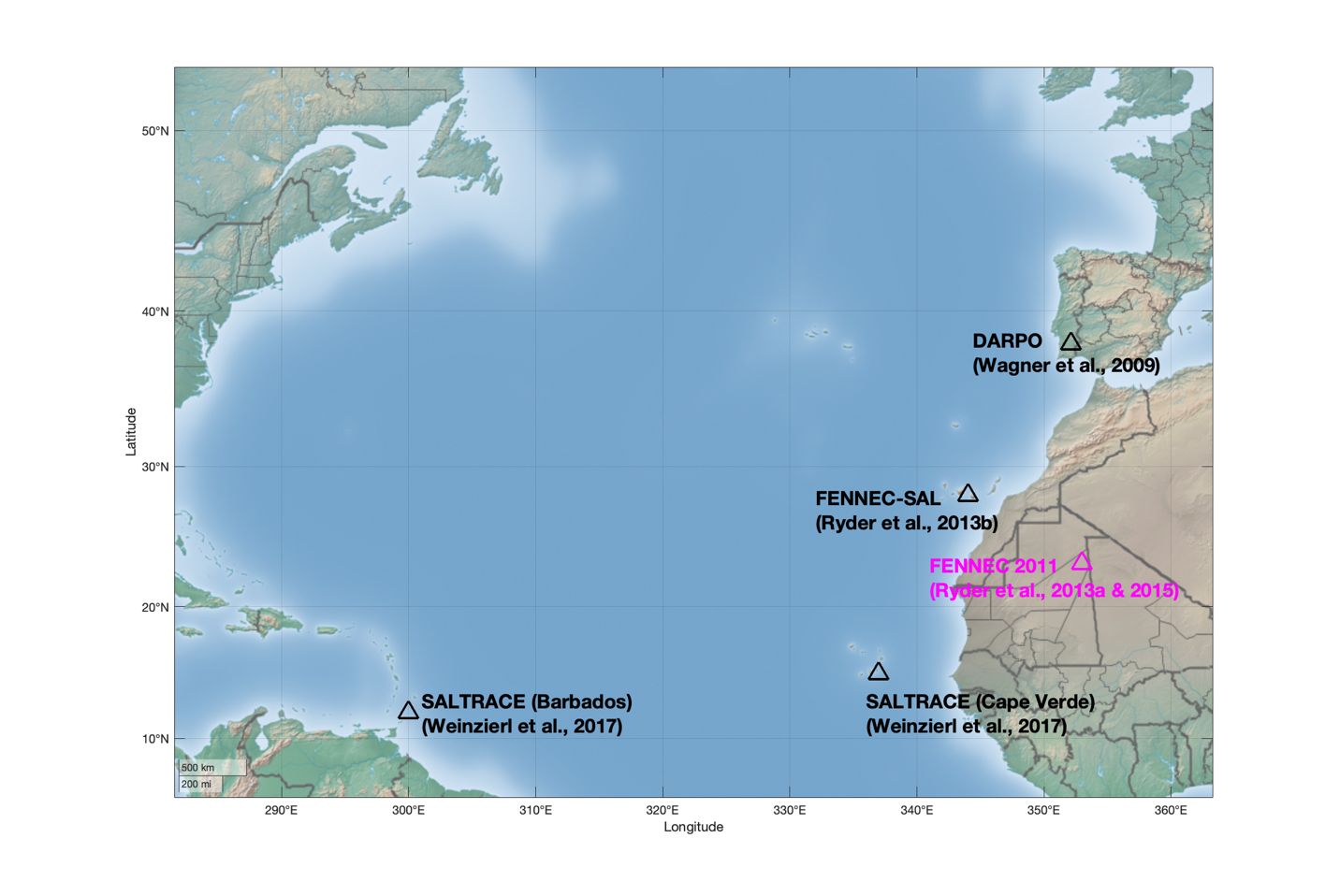
**Figure S2.** **Locations of atmospheric PSD measurements campaigns used in this study.** Aircraft campaigns conducted in or close to the dust source regions are denoted with pink triangles and campaigns conducted in desert outflow regions are denoted with black triangles.


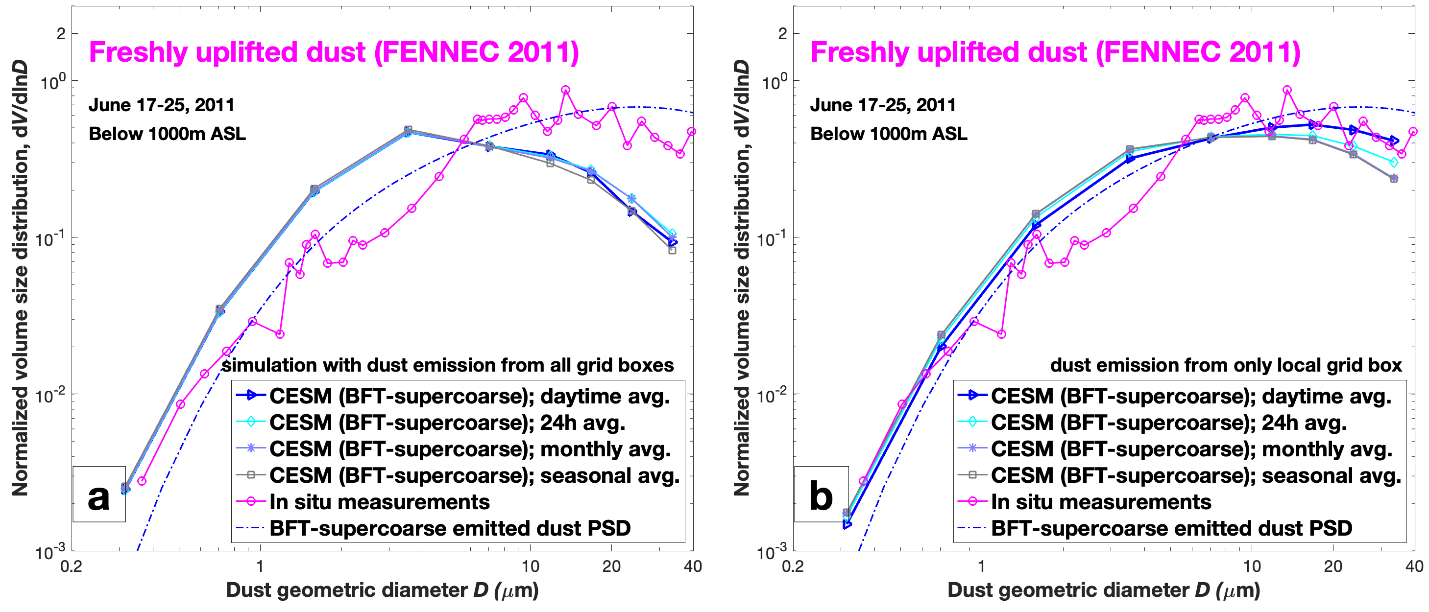


**Figure S3. Simulations showing the sensitivity of the simulated freshly uplifted atmospheric dust PSD to whether dust emissions are included from all grid boxes or just the grid box identified as the dust source and the simulated atmospheric PSD averaged at different temporal resolutions.** Shown are the simulated PSDs using BFT-supercoarse when averaged over daytime hours only (10 - 18h local time) (blue line with triangle markers), the entire day (cyan line with diamond markers), the entire month (light blue line with asterisk markers) and the entire season (grey line with square markers). The magenta open circles denote the average PSD of freshly uplifted dust over Mali and Maritania (~23.5°N, 7°W) during FENNEC campaign flights B600, B601, B602 and B610 at altitudes below 1000 m. In (a), simulations are with BFT-supercoarse and emissions from all model grid-boxes. In (b), simulations are with BFT-supercoarse but with emissions only at the grid-box where the freshly uplifted dust observations (Flights B600, B601, B602 and B610) were taken, which was identified as the source of the sampled dust. This figure indicates that the effect of the temporal scale over which model simulations of the PSD are averaged is relatively small. All curves are normalized to yield unity when integrated over the 0.1-20 μm diameter range.


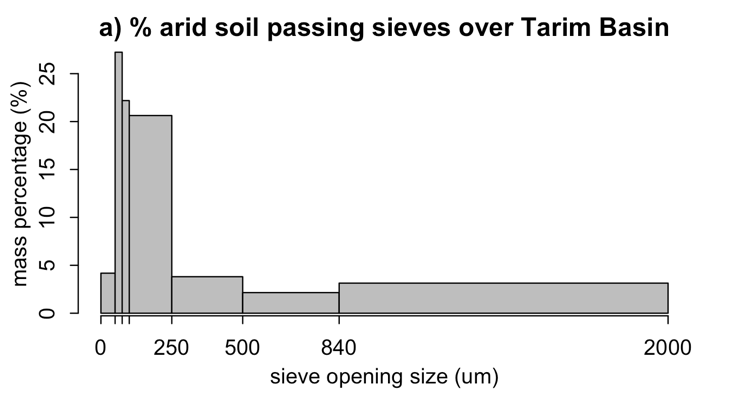


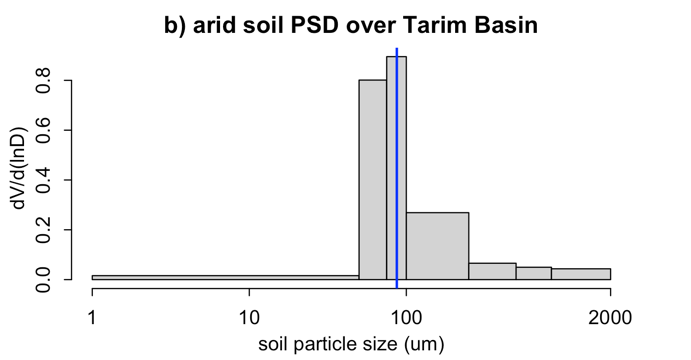

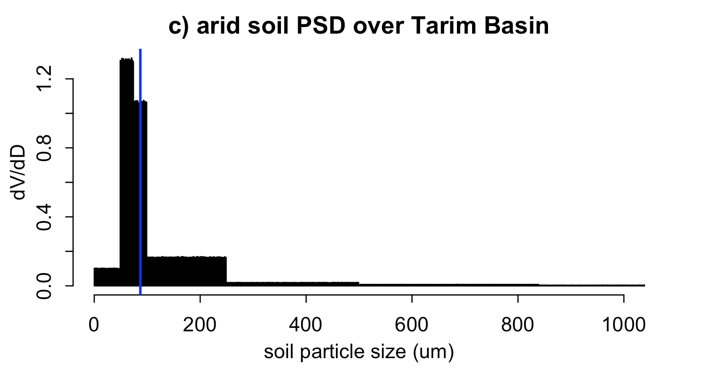


**Figure S4.** **An example of a soil particle size distribution (PSD) for an arid soil in the Tarim Basin from Li et al. (2014).** (a) The mass percentage of soil particles passing through different sieve openings (x-axis) was obtained from Table 3 in Li et al. (2014) and documented in our Table S1. (b) The soil PSD displayed as $dV/dlnD$ versus $D$, where $V$ is soil volume and $\ln D$ is the natural logarithm of particle diameter $D$. The blue line indicates the median diameter $D$*,* determined by dividing the percentages from (a) by their respective bin widths, dividing this into sub-bins of width 1 μm (panel c), and identifying the sub-bin at which the cumulative mass fraction reaches 50%. The median diameter is $D=87 \mu m$ for this case. Note that the area in panels (b) and (c) sum up to 1.


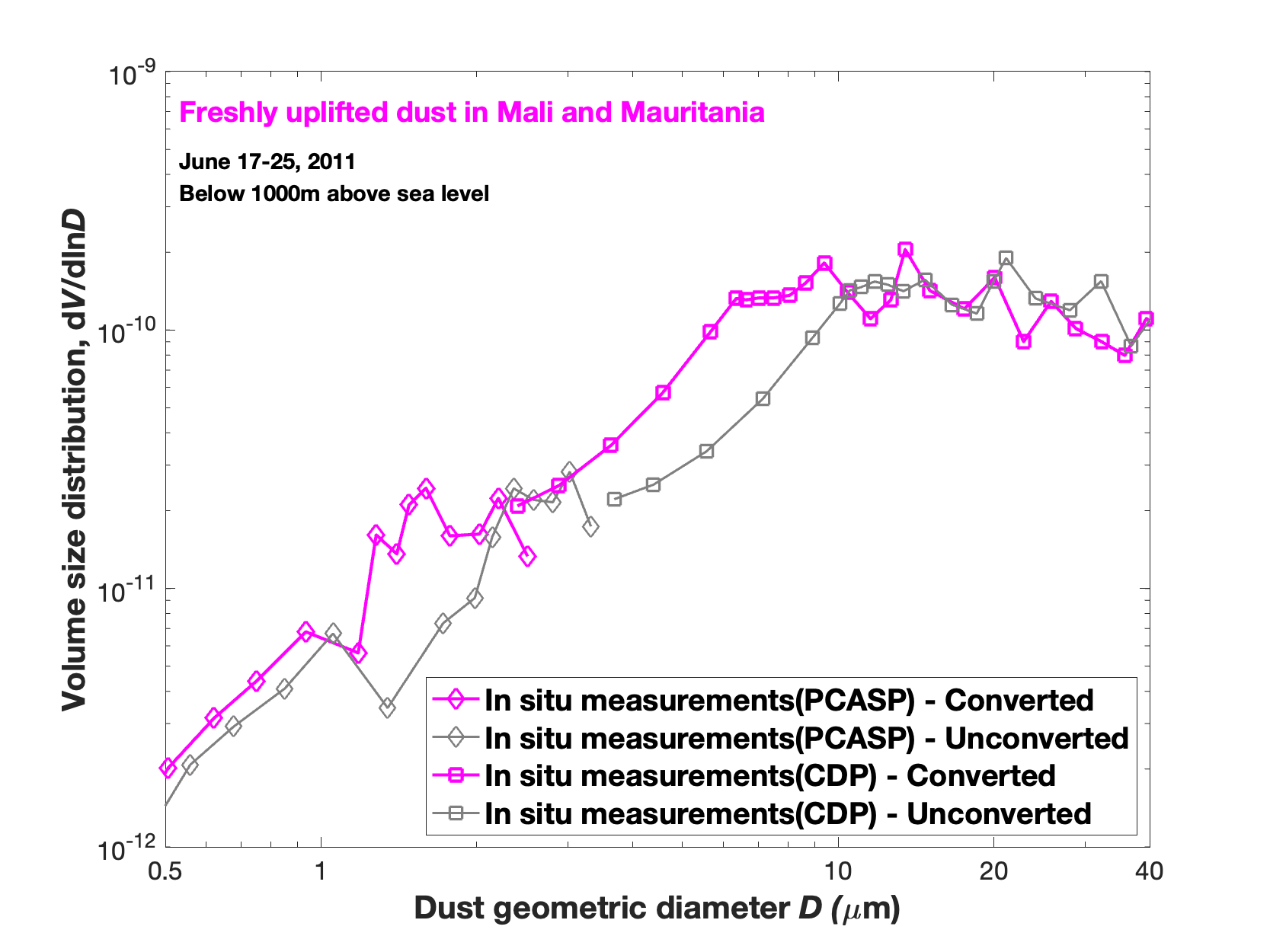


**Figure S5. Effect of accounting for asphericity on FENNEC PSD measurements for FENNEC flight-averaged PSD measurements (flights B600, B601, B602 and B610).** The conversion for dust shape was done following Huang et al. (2021). The magenta line is the converted data, which including the correction for dust refractive index and dust shape. The grey line is the unconverted data, which only including the correction for dust refractive index; the square markers denote the measurements from the CDP instrument whereas the diamond markers denote the measurements from the PCASP instrument.


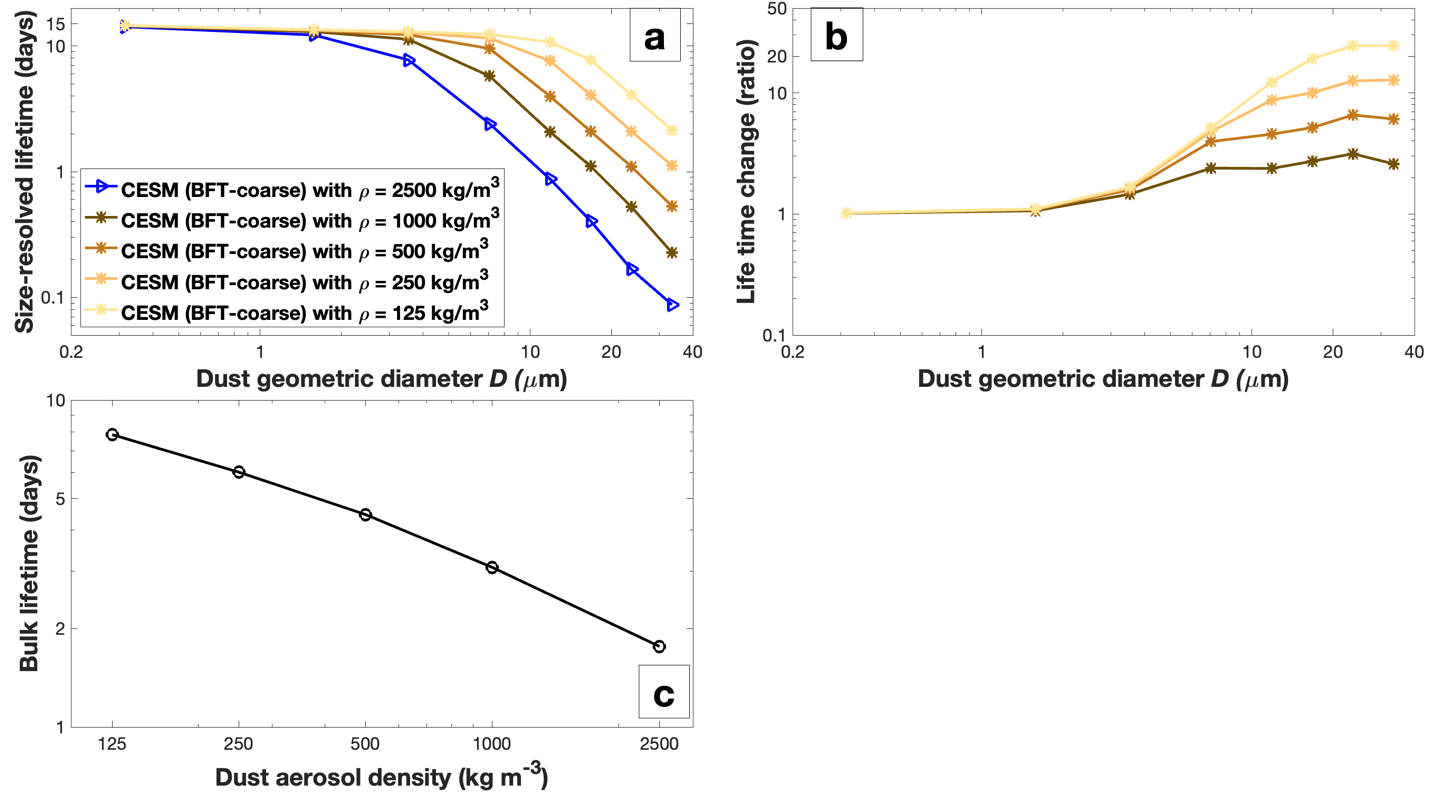


**Figure S6. Simulated atmospheric dust lifetime.** (a). Shown are size-resolved dust lifetime from simulations using the default dust aerosol density (2500 kg m^-3^, blue line with triangle markers) and four sensitivity simulations using smaller dust aerosol densities (1000 kg m^-3^, dark brown line; 500 kg m^-3^, brown line; 250 kg m^-3^, orange line; 125 kg m^-3^, light yellow line); all simulations use the BFT-supercoarse emitted dust PSD. (b). The size-resolved dust lifetime changes for simulations using lower dust aerosol densities compared with the simulation using the default dust aerosol density. (c). Bulk global averaged dust lifetime from simulations using different dust aerosol densities.

*
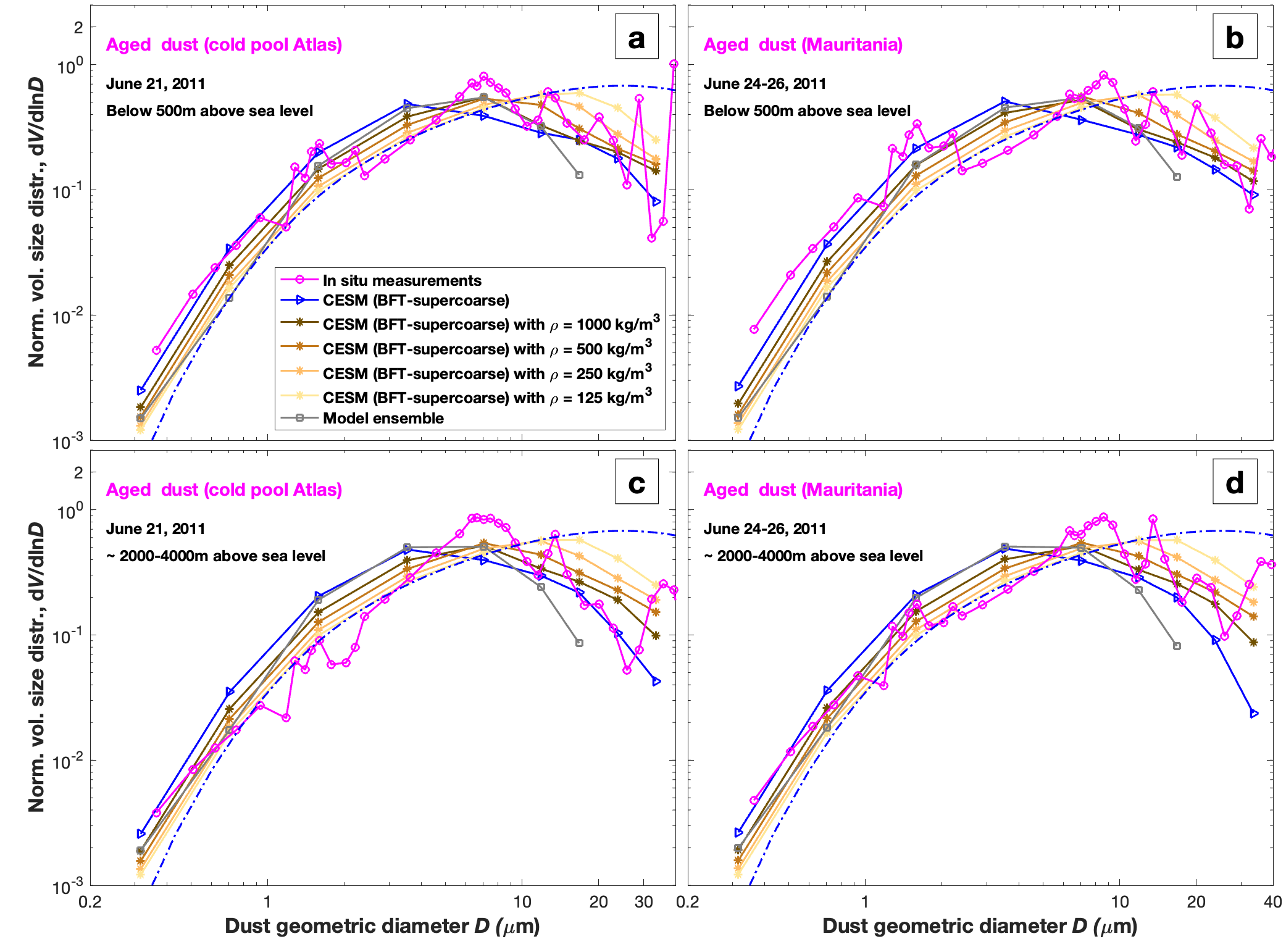
*

**Figure S7. Comparison between simulations with reduced dust density and measurements of the dust PSD close to dust source regions.** Simulated atmospheric dust PSDs include a simulation using the default dust aerosol density (2500 kg m^-3^, blue line with triangle markers) and four simulations using smaller dust aerosol densities (1000 kg m^-3^, dark brown line; 500 kg m^-3^, brown line; 250 kg m^-3^, orange line; 125 kg m^-3^, light yellow line); all simulations use the BFT-supercoarse emitted dust PSD. The gray line is the seasonally averaged dust PSD from an ensemble of model simulations (Adebiyi et al., 2020). The blue dash-dotted line denotes the parameterization of emitted dust developed in this study (BFT-supercoarse). Atmospheric dust PSD measurements (magenta open circles) are from FENNEC 2011 flights that measured aged dust events near dust source regions, including aged dust from a cold pool near the Atlas mountains (flights B605 and B606) and from Mauritania (flights B609, B611-613) (Ryder et al., 2015). Locations of the measurements are shown in Figure S2. All curves are normalized to yield unity when integrated over the 0.1-20 μm diameter range.

**References**

Åström, J. A. (2006). Statistical models of brittle fragmentation. *Advances in Physics*, *55*(3–4), 247–278. <https://doi.org/10.1080/00018730600731907>

Chandler, D. G., Saxton, K. E., & Busacca, A. J. (2004). Predicting Wind Erodibility of Loessial Soils in the Pacific Northwest by Particle Sizing. *Arid Land Research and Management*, *19*(1), 13–27. https://doi.org/10.1080/15324980590887074

Chatenet, B., Marticorena, B., Gomes, L., & Bergametti, G. (1996). Assessing the microped size distributions of desert soils erodible by wind. *Sedimentology*, *43*(5), 901–911. https://doi.org/10.1111/j.1365-3091.1996.tb01509.x

Gelaro, R., McCarty, W., Suárez, M. J., Todling, R., Molod, A., Takacs, L., et al. (2017). The Modern-Era Retrospective Analysis for Research and Applications, Version 2 (MERRA-2). *Journal of Climate*, *30*(14), 5419–5454. https://doi.org/10.1175/JCLI-D-16-0758.1

Huang, Y., Kok, J. F., Martin, R. L., Swet, N., Katra, I., Gill, T. E., et al. (2019). Fine dust emissions from active sands at coastal Oceano Dunes, California. *Atmospheric Chemistry and Physics*, *19*(5), 2947–2964. https://doi.org/10.5194/acp-19-2947-2019

Huang, Y., Adebiyi, A. A., Formenti, P., & Kok, J. F. (2021). Linking the Different Diameter Types of Aspherical Desert Dust Indicates That Models Underestimate Coarse Dust Emission. *Geophysical Research Letters*, *48*(6), e2020GL092054. https://doi.org/10.1029/2020GL092054

Hurrell, J. W., Holland, M. M., Gent, P. R., Ghan, S., Kay, J. E., Kushner, P. J., et al. (2013). The Community Earth System Model: A Framework for Collaborative Research. *Bulletin of the American Meteorological Society*, *94*(9), 1339–1360. https://doi.org/10.1175/BAMS-D-12-00121.1

ISO. (2009). Determination of particle size distribution — Single particle light interaction methods — Part 1: Light scattering aerosol spectrometer. Retrieved September 14, 2021, from https://www.iso.org/cms/render/live/en/sites/isoorg/contents/data/standard/04/27/42728.html

Klose, M., & Shao, Y. (2012). Stochastic parameterization of dust emission and application to convective atmospheric conditions. *Atmospheric Chemistry and Physics*, *12*(16), 7309–7320. https://doi.org/10.5194/acp-12-7309-2012

Klose, Martina, Gill, T. E., Webb, N. P., & Van Zee, J. W. (2017). Field sampling of loose erodible material: A new system to consider the full particle-size spectrum. *Aeolian Research*, *28*, 83–90. https://doi.org/10.1016/j.aeolia.2017.08.003

Kok, J. F. (2011a). A scaling theory for the size distribution of emitted dust aerosols suggests climate models underestimate the size of the global dust cycle. *Proceedings of the National Academy of Sciences*, *108*(3), 1016–1021. https://doi.org/10.1073/pnas.1014798108

Kok, J. F. (2011b). Does the size distribution of mineral dust aerosols depend on the wind speed at emission? *Atmospheric Chemistry and Physics*, *11*(19), 10149–10156. https://doi.org/10.5194/acp-11-10149-2011

Kok, J. F., Mahowald, N. M., Fratini, G., Gillies, J. A., Ishizuka, M., Leys, J. F., et al. (2014). An improved dust emission model – Part 1: Model description and comparison against measurements. *Atmospheric Chemistry and Physics*, *14*(23), 13023–13041. https://doi.org/10.5194/acp-14-13023-2014

Kok, J. F., Albani, S., Mahowald, N. M., & Ward, D. S. (2014). An improved dust emission model – Part 2: Evaluation in the Community Earth System Model, with implications for the use of dust source functions. *Atmospheric Chemistry and Physics*, *14*(23), 13043–13061. https://doi.org/10.5194/acp-14-13043-2014

Kok, J. F., Ridley, D. A., Zhou, Q., Miller, R. L., Zhao, C., Heald, C. L., et al. (2017). Smaller desert dust cooling effect estimated from analysis of dust size and abundance. *Nature Geoscience*, *10*(4), 274–278. https://doi.org/10.1038/ngeo2912

Lawrence, D. M., Oleson, K. W., Flanner, M. G., Thornton, P. E., Swenson, S. C., Lawrence, P. J., et al. (2011). Parameterization improvements and functional and structural advances in Version 4 of the Community Land Model. *Journal of Advances in Modeling Earth Systems*, *3*(1). https://doi.org/10.1029/2011MS00045

Leung, D. M., Kok, J. F., Li, L., Mahowald, N. M., Menut, L., Prigent, C., et al. (2020). Improving the parameterization of dust emission threshold in the Community Earth System Model (CESM), *2020*, A041-0012. Presented at the AGU Fall Meeting Abstracts. https://doi.org/10.1002/essoar.10506234.1

Li, X., Feng, G., Sharratt, B. S., Zheng, Z., Pi, H., & Gao, F. (2014). Soil Wind Erodibility Based on Dry Aggregate-Size Distribution in the Tarim Basin. *Soil Science Society of America Journal*, *78*(6), 2009–2016. https://doi.org/10.2136/sssaj2014.06.0235

Liu, L., Wang, J., Li, X., Liu, Y., Ta, W., & Peng, H. (1998). Determination of erodible particles on cultivated soils by wind tunnel simulation. *Chinese Science Bulletin*, *43*(19), 1646–1651. https://doi.org/10.1007/BF02883411

Mahowald, N., Albani, S., Kok, J. F., Engelstaeder, S., Scanza, R., Ward, D. S., & Flanner, M. G. (2014). The size distribution of desert dust aerosols and its impact on the Earth system. *Aeolian Research*, *15*, 53–71. https://doi.org/10.1016/j.aeolia.2013.09.002

Mahowald, N. M., Muhs, D. R., Levis, S., Rasch, P. J., Yoshioka, M., Zender, C. S., & Luo, C. (2006). Change in atmospheric mineral aerosols in response to climate: Last glacial period, preindustrial, modern, and doubled carbon dioxide climates. *Journal of Geophysical Research: Atmospheres*, *111*(D10). https://doi.org/10.1029/2005JD006653

Mei, F., Zhang, X., Lu, H., Shen, Z., & Wang, Y. (2004). Characterization of MASDs of surface soils in north China and its influence on estimating dust emission. *Chinese Science Bulletin*, *49*(20), 2169–2176. https://doi.org/10.1007/BF03185784

Meng, Z., Yang, P., Kattawar, G. W., Bi, L., Liou, K. N., & Laszlo, I. (2010). Single-scattering properties of tri-axial ellipsoidal mineral dust aerosols: A database for application to radiative transfer calculations. *Journal of Aerosol Science*, *41*(5), 501–512. https://doi.org/10.1016/j.jaerosci.2010.02.008

Neale, R. B., Richter, J. H., Conley, A. J., Park, S., Lauritzen, P. H., Gettelman, A., et al. (2010). *Description of the NCAR Community Atmosphere Model*.

Rosenberg, P. D., Dean, A. R., Williams, P. I., Dorsey, J. R., Minikin, A., Pickering, M. A., & Petzold, A. (2012). Particle sizing calibration with refractive index correction for light scattering optical particle counters and impacts upon PCASP and CDP data collected during the Fennec campaign. *Atmospheric Measurement Techniques*, *5*(5), 1147–1163. https://doi.org/10.5194/amt-5-1147-2012

Ryder, C. L., Highwood, E. J., Lai, T. M., Sodemann, H., & Marsham, J. H. (2013). Impact of atmospheric transport on the evolution of microphysical and optical properties of Saharan dust. *Geophysical Research Letters*, *40*(10), 2433–2438. https://doi.org/10.1002/grl.50482

Ryder, C. L., Highwood, E. J., Rosenberg, P. D., Trembath, J., Brooke, J. K., Bart, M., et al. (2013). Optical properties of Saharan dust aerosol and contribution from the coarse mode as measured during the Fennec 2011 aircraft campaign. *Atmospheric Chemistry and Physics*, *13*(1), 303–325. https://doi.org/10.5194/acp-13-303-2013

Ryder, C. L., McQuaid, J. B., Flamant, C., Rosenberg, P. D., Washington, R., Brindley, H. E., et al. (2015). Advances in understanding mineral dust and boundary layer processes over the Sahara from Fennec aircraft observations. *Atmospheric Chemistry and Physics*, *15*(14), 8479–8520. https://doi.org/10.5194/acp-15-8479-2015

Shao, Y., Ishizuka, M., Mikami, M., & Leys, J. F. (2011). Parameterization of size-resolved dust emission and validation with measurements. *Journal of Geophysical Research: Atmospheres*, *116*(D8). https://doi.org/10.1029/2010JD014527

Shao, Y., Zhang, J., Ishizuka, M., Mikami, M., Leys, J., & Huang, N. (2020). Dependency of particle size distribution at dust emission on friction velocity and atmospheric boundary-layer stability. *Atmospheric Chemistry and Physics*, *20*(21), 12939–12953. https://doi.org/10.5194/acp-20-12939-2020

Su, Y., Wang, F., Zhang, Z., & Du, M. (2007). Soil Properties and Characteristics of Soil Aggregate in Marginal Farmlands of Oasis in the Middle of Hexi Corridor Region, Northwest China. *Agricultural Sciences in China*, *6*(6), 706–714. https://doi.org/10.1016/S1671-2927(07)60103-5

Swet, N., & Katra, I. (2016). Reduction in soil aggregation in response to dust emission processes. *Geomorphology*, *268*, 177–183. https://doi.org/10.1016/j.geomorph.2016.06.002

Wagner, F., Bortoli, D., Pereira, S., Costa, M. Jo., Silva, A. M., Weinzierl, B., et al. (2009). Properties of dust aerosol particles transported to Portugal from the Sahara desert. *Tellus B: Chemical and Physical Meteorology*, *61*(1), 297–306. https://doi.org/10.1111/j.1600-0889.2008.00393.x

Weinzierl, B., Petzold, A., Esselborn, M., Wirth, M., Rasp, K., Kandler, K., et al. (2009). Airborne measurements of dust layer properties, particle size distribution and mixing state of Saharan dust during SAMUM 2006. *Tellus B: Chemical and Physical Meteorology*, *61*(1), 96–117. https://doi.org/10.1111/j.1600-0889.2008.00392.x

Weinzierl, B., Ansmann, A., Prospero, J. M., Althausen, D., Benker, N., Chouza, F., et al. (2017). The Saharan Aerosol Long-Range Transport and Aerosol–Cloud-Interaction Experiment: Overview and Selected Highlights. *Bulletin of the American Meteorological Society*, *98*(7), 1427–1451. https://doi.org/10.1175/BAMS-D-15-00142.1

Zender, C. S., Bian, H., & Newman, D. (2003). Mineral Dust Entrainment and Deposition (DEAD) model: Description and 1990s dust climatology. *Journal of Geophysical Research: Atmospheres*, *108*(D14). https://doi.org/10.1029/2002JD002775

Fratini, G., Ciccioli, P., Febo, A., Forgione, A., & Valentini, R. (2007). Size-segregated fluxes of mineral dust from a desert area of northern China by eddy covariance. Atmospheric Chemistry and Physics, 7(11), 2133–2168. https://doi.org/10.5194/acpd-7-2133-2007

Gillette, D. A. (1974). On the production of soil wind erosion aerosols having the potential for long range transport. Journal de Recherches Atmospheriques, 8, 734–744.

Gillette, D. A., Blifford, I. H., Jr., & Fenster, C. R. (1972). Measurements of aerosol size distributions and vertical fluxes of aerosols on land subject to wind erosion. Journal of Applied Meteorology, 11(6), 977–987. [https://doi.org/10.1175/1520-0450(1972)011<0977:MOASDA>2.0.CO;2](https://doi.org/10.1175/1520-0450(1972)011%3c0977:MOASDA%3e2.0.CO;2)

Gillette, D. A., Blifford, I. H., Jr., & Fryrear, D. W. (1974). The influence of wind velocity on the size distributions of aerosols generated by the wind erosion of soils. Journal of Geophysical Research, 79(27), 4068–4075. <https://doi.org/10.1029/JC079i027p04068>

Khalfallah, B., Bouet, C., Labiadh, M. T., Alfaro, S. C., Bergametti, G., Marticorena, B., et al. (2020). Influence of atmospheric stability on the size distribution of the vertical dust flux measured in eroding conditions over a flat bare sandy field. Journal of Geophysical Research: Atmospheres, 125(4), 1–20. <https://doi.org/10.1029/2019JD031185>

Rosenberg, P. D., Parker, D. J., Ryder, C. L., Marsham, J. H., Garcia-Carreras, L., Dorsey, J. R., et al. (2014). Quantifying particle size and turbulent scale dependence of dust flux in the Sahara using aircraft measurements. Journal of Geophysical Research: Atmospheres, 119(12), 7577–7598. <https://doi.org/10.1002/2013JD021255>

Sow, M., Alfaro, S. C., Rajot, J. L., & Marticorena, B. (2009). Size resolved dust emission fluxes measured in Niger during 3 dust storms of the AMMA experiment. Atmospheric Chemistry and Physics, 9(12), 3881–3891. <https://doi.org/10.5194/acp-9-3881-2009>
